# Psychometric Properties of the SarQoL Questionnaire: A Systematic Review and Meta‐Analysis

**DOI:** 10.1002/jcsm.70122

**Published:** 2025-11-23

**Authors:** Céline Demonceau, Brabant Christian, Shukuru Emmanuel, Majed Alokail, Nasser Al‐Daghri, Yves Rolland, Ivan Bautmans, Jürgen M. Bauer, Antonio Cherubini, Alfonso J. Cruz‐Jentoft, Bess Dawson‐Hughes, Roger A. Fielding, Nicholas C. Harvey, Francesco Landi, Marjolein Visser, Gustavo Duque, René Rizzoli, Jean‐Yves Reginster, Olivier Bruyère, Charlotte Beaudart

**Affiliations:** ^1^ Research Unit in Public Health, Epidemiology and Health Economics University of Liège Liège Belgium; ^2^ Public Health Aging Research & Epidemiology (PHARE)Group, Research Unit in Clinical Pharmacologyand Toxicology (URPC), Department of Biomedical Sciences‑Faculty of Medicine, NAmur Research Institute for LIfeSciences (NARILIS) University of Namur Namur Belgium; ^3^ Protein Research Chair, Biochemistry Department, College of Science King Saud University Riyadh Kingdom of Saudi Arabia; ^4^ IHU HealthAge, Gérontopôle de Toulouse, Institut du Vieillissement Centre Hospitalo‐Universitaire de Toulouse Toulouse France; ^5^ Gerontology Department and Frailty in Ageing Research Department Vrije Universiteit Brussel Brussels Belgium; ^6^ Center for Geriatric Medicine and Network Aging Research (NAR) Heidelberg University Heidelberg Germany; ^7^ Geriatria, Accettazione Geriatrica e Centro di Ricerca per l'Invecchiamento IRCCS INRCA Ancona Italy; ^8^ Department of Clinical and Molecular Sciences Università Politecnica delle Marche Ancona Italy; ^9^ Servicio de Geriatría Hospital Universitario Ramón y Cajal (IRYCIS) Madrid Spain; ^10^ Jean Mayer USDA Human Nutrition Research Center on Aging Tufts University Boston Massachusetts USA; ^11^ Nutrition, Exercise Physiology and Sarcopenia Laboratory, Jean Mayer USDA Human Nutrition Research Centeron Aging Tufts University Boston Massachusetts USA; ^12^ MRC Lifecourse Epidemiology Centre University of Southampton Southampton UK; ^13^ NIHR Southampton Biomedical Research Centre University of Southampton Southampton UK; ^14^ Fondazione Policlinico Universitario “Agostino Gemelli” IRCCS Rome Italy; ^15^ Department of Health Sciences, Faculty of Science Vrije Universiteit Amsterdam and the Amsterdam Public Health Research Institute Amsterdam the Netherlands; ^16^ Bone, Muscle & Geroscience Group Research Institute of the McGill University Health Centre Montreal Quebec Canada; ^17^ Department of Medicine McGill University Montreal Quebec Canada; ^18^ Service of Bone Diseases, Faculty of Medicine Geneva University Hospitals Geneva Switzerland

**Keywords:** meta‐analysis, PROMs, psychometric properties, quality of life, sarcopenia, SarQoL

## Abstract

**Background:**

The Sarcopenia and Quality of Life (SarQoL) questionnaire is recognized as the only disease‐specific patient‐reported outcome measure (PROM) for assessing sarcopenia‐related HRQoL. This systematic review and meta‐analysis aimed to provide a quantitative summary of all evidence reported on the reliability, validity, responsiveness and floor/ceiling effects of SarQoL in older adults.

**Methods:**

Following PRISMA‐COSMIN guidelines, a systematic search for studies evaluating the psychometric properties of SarQoL (i.e., reliability, validity, responsiveness and floor and ceiling effects) in older people was conducted on MEDLINE (via OVID), PsycINFO, Scopus and EMBASE. Studies published between 2013 and November 2024 using a consensual definition of sarcopenia were included. Study selection and data extraction were made by two independent reviewers. A random‐effects model meta‐analysis was applied. PROSPERO registration: CRD42024546880.

**Results:**

From 411 studies identified by the search strategy, 25 fulfilled the inclusion criteria, including 4585 community‐dwelling individuals, of which 1311 were diagnosed as sarcopenic. SarQoL demonstrated high reliability (pooled Cronbach's alpha values consistently exceeding 0.80) and excellent test–retest reliability (pooled ICC = 0.98). Construct validity was confirmed with strong convergent correlations (pooled *r* > 0.54) with related dimensions of generic SF‐36 and EQ‐5D and weaker divergent correlations (pooled *r* < 0.47). Responsiveness, evaluated in two studies using different methodologies, supported the ability of SarQoL to detect meaningful changes in HRQoL. The certainty of evidence was rated as high for reliability, validity and responsiveness.

**Conclusion:**

This meta‐analysis consolidates a decade of evidence and confirms the strong psychometric properties of SarQoL, with a high level of evidence.

## 1 Introduction

Sarcopenia is a common geriatric condition characterized by a loss of muscle mass and strength, the severity of which is reflected by poor physical performance [1]. A recent meta‐analysis estimated the prevalence of sarcopenia to be between 10% and 16% among older adults, recognizing this condition as a major public health concern [2]. Indeed, numerous studies have highlighted the detrimental impact of sarcopenia on falls, fractures, hospitalization, mortality and health‐related quality of life (HRQoL) [2]. Beyond its well‐documented physical and functional consequences, sarcopenia has also been associated with a significant decline in health‐related quality of life (HRQoL), notably through its impact on autonomy, mobility and overall functioning [3]. In this regard, recent international recommendations, including those of the working group of the Global Leadership Initiative in Sarcopenia (GLIS), underscore the importance of considering HRQoL as a key outcome in both clinical assessment and research protocols targeting sarcopenia [4].

HRQoL, defined by the World Health Organization (WHO) as the way individuals view themselves, their place in life, their cultural and value systems and how these align with their aspirations, concerns, perspectives and personal standards [5], is considered a complex and multidimensional concept that can be challenging to define and measure accurately [6]. In this context, patient‐reported outcome measures (PROMs) are valuable tools to capture the subjective aspects of health, especially in the context of specific diseases [7]. Until 2013, only generic tools were available to assess HRQoL in individuals with sarcopenia. To address this gap, the Sarcopenia and Quality of Life (SarQoL) questionnaire was developed to provide a disease‐specific measure of HRQoL [8].

This validated questionnaire consists of 22 questions across seven dimensions that capture critical aspects of life affected by sarcopenia, including physical and mental health, locomotion, body composition, functionality, activities of daily living, leisure activities and fears. It provides a composite score out of 100, providing a comprehensive and holistic reflection of HRQoL [9].

Since its development, SarQoL has gained widespread recognition as the only disease‐specific tool for assessing HRQoL in sarcopenia, with translations available in more than 30 languages worldwide (available at: https://sarqol.org/en/sarqol_form). A recent meta‐analysis has highlighted the superior discriminatory power of SarQoL to assess HRQoL in sarcopenic individuals compared to generic tools such as SF‐36 or EQ‐5D questionnaires, underscoring its relevance in clinical and research settings for the sarcopenic population [3].

Given the increasing use of the SarQoL questionnaire in both research and clinical settings, it is essential to ensure that its psychometric properties, including its reliability, validity and responsiveness, are rigorously evaluated in order to confirm its relevance for measuring QoL in older adults with sarcopenia. While many individual studies have reported on these properties, pooling the available evidence within a single statistical model allows for a more comprehensive understanding of the overall performance of the instrument.

PROMs, such as SarQoL, require robust psychometric evaluation to ensure that they accurately reflect HRQoL in people with sarcopenia. According to COSMIN guidance, these evaluations encompass key psychometric properties, such as reliability, validity and responsiveness [10]. While numerous individual studies have reported strong evidence supporting these properties since the initial validation of SarQoL [9], the absence of a comprehensive quantitative synthesis limits the ability to confirm these findings across different populations and settings. Although a recent review of the literature on the psychometric properties of SarQoL provided valuable insights [11], it did not include a meta‐analysis to quantitatively synthesize the findings. In addition, since the publication of this review, new evidence on the psychometric properties of SarQoL has emerged, such as a recent publication reporting the content validity of the SarQoL questionnaire that has been published [12]. This evolving body of research provides a robust basis for the first meta‐analytic approach to consolidate the existing evidence on the psychometric properties of SarQoL, using a COSMIN‐based categorization of these properties and incorporating the most recent level of evidence available in the field. This enables a more comprehensive and generalizable assessment of the reliability, validity and responsiveness of SarQoL in older adults.

## 2 Methods

### 2.1 Study Selection

A protocol has been developed and registered on PROSPERO (CRD42024546880). The objective of this systematic review and meta‐analysis was to provide a comprehensive synthesis of the psychometric properties of the SarQoL questionnaire, including its reliability, construct and content validity, responsiveness and the presence of floor or ceiling effects. Specifically, the present study aimed to summarize the available evidence quantitatively, using meta‐analytical methods, assess its methodological quality and apply the GRADE approach to evaluate the certainty of the evidence. The 2024 updated PRISMA‐COSMIN for Outcome Measurement Instruments was followed for all steps of this research [13]. The completed relevant checklist can be found in Appendix S1.

### 2.2 Search Strategy and Selection Criteria

Systematic searches on MEDLINE (via OVID platform), PsycINFO, Scopus and EMBASE databases were conducted on 1 March 2024 to identify studies reporting on the psychometric properties of SarQoL, with an update performed in December 2024. The search strategy (Appendix S2) aimed to identify original studies published in English between November 2013 (i.e., the date of SarQoL development) and December 2024. In addition, manual searches of the references of relevant papers were undertaken to identify potential additional references. As the research team included experts in the field of sarcopenia and the development of SarQoL, we used their expertise to minimize the risk of missing relevant studies.

References from both electronic and manual searches have been imported into Covidence software [14]. Two independent reviewers have assessed the eligibility of all identified articles based on the defined inclusion criteria listed in Table 1. The screening was first based on the title and abstract, followed by a second screening based on the full text. A third reviewer was consulted in case of disagreement. Each screening step has been reported and presented in a PRISMA flowchart.

**TABLE 1:** Inclusion criteria.

Participants	Sarcopenic individuals aged 60 years or older (mean or median age of the sample) living in the community or in assisted living facilities are eligible for inclusion.
Sarcopenia definition	The diagnosis of sarcopenia should include at least two biomarkers: lean mass or muscle mass in combination with either muscle strength or physical function using a consensual definition of sarcopenia (e.g., EWGSOP1, EWGSOP2, AWGS, etc.).
Outcome	Only studies reporting the following properties of SarQoL were included: Reliability: Internal validity measured by Cronbach's/Omega's alpha Test–retest reliability measured by Intraclass Correlation Coefficients (ICC) Measurement error measured by the standard error of measurement (SEM) Validity: Convergent/divergent validity measured by Spearman's/Pearson's correlations Content validity through relevance, comprehensiveness and comprehensibility Responsiveness: By distribution or anchor‐based methods Floor and ceiling effects.
Study design	Observational studies (i.e., cross‐sectional or longitudinal) providing original data.
Language	English [13]

Data from relevant studies have been extracted by two reviewers and recorded in an Excel spreadsheet. The following information has been extracted in a standardized form: article information (e.g., author names, correspondence email, study title, year of publication, continent, country), population characteristics (e.g., age, gender, sarcopenia diagnosis), outcomes (reliability, validity and responsiveness and floor/ceiling effect), funding and conflicts of interest.

### 2.3 Quality Appraisal

The quality of the studies was assessed using the COSMIN risk of bias checklist. This tool rates each measurement property from very good to inadequate according to predefined criteria and the presence of methodological flaws. Two reviewers independently assessed the risk of bias (RoB) for each psychometric property of each study using the COSMIN recommendations [7]. Disagreements were resolved by consensus or by a third reviewer.

### 2.4 Psychometric Properties

According to the COSMIN taxonomy, SarQoL has been assessed according to three quality domains (i.e., reliability, validity and responsiveness), each including one or more measurement properties [15].

#### 2.4.1 Reliability

Reliability corresponds to the extent to which the patient's score has not changed during a specified period of time under the same test administration conditions [15].

The reliability of SarQoL was assessed using three different measurement properties: internal consistency, test–retest reliability and measurement error.

Internal consistency, which is an estimate of item homogeneity, is measured using the Cronbach alpha coefficient, and a value greater than 0.7 is recommended to be considered adequate [16]. In addition, the impact of each domain on reliability can be tested by correlating each domain score with the global SarQoL score using Spearman or Pearson correlation. A correlation above 0.81 is considered excellent, between 0.61 and 0.80 as very good, between 0.41 and 0.60 as good, between 0.21 and 0.4 as acceptable and, finally, less than 0.20 as insufficient [17].

Test–retest reliability corresponds to the correlation of the questionnaire scores when it is administered over a period of time measured by an intraclass coefficient correlation (ICC) between the scores (global and for each domain). An ICC greater than 0.7 is recommended to be considered adequate reliability [18].

Finally, measurement error, defined as the variation in the patient's score due to systematic and random error rather than changes in the construct being measured, is reported using standard error of measurement (SEM) or smallest detectable change (SDC) [15].

#### 2.4.2 Validity

Validity is defined as the extent to which a questionnaire measures the construct it is intended to measure. Two measurement properties were used to assess the validity of SarQoL: construct validity and content validity [15].

Based on COSMIN recommendations, the construct validity of SarQoL was assessed using a priori hypotheses regarding two other generic questionnaires sometimes used to measure HRQoL: the Short‐Form‐36 (SF‐36) and the EuroQoL 5‐Dimension (EQ‐5D) questionnaires [19, 20]. Convergent and divergent validity were assessed between SarQoL dimensions and corresponding dimensions using Spearman or Pearson correlations to determine the effectiveness of SarQoL in accurately measuring the intended construct [15].

Content validity refers to the extent to which the content of an instrument accurately reflects the construct being measured. According to the COSMIN methodology, three aspects should be examined to assess the content validity in patients and experts: relevance, comprehensiveness and comprehensibility [21].

#### 2.4.3 Responsiveness

Responsiveness is the ability of a PROMs to capture change, to detect clinically important changes in the measured construct over time, including small changes, and can be compared for longitudinal validity [18]. Responsiveness can be measured using several methods, such as the effect size (ES) of a receiver operator characteristic (ROC) curve [22].

In addition to reliability, validity and responsiveness, we have also considered floor and ceiling effects as quality criteria for SarQoL. However, they are not considered psychometric properties in the COSMIN taxonomy. Floor or ceiling effects are present when more than 15% of respondents score at the lowest or highest possible level [18].

#### 2.4.4 Certainty Assessment

The certainty of the evidence for reliability, validity and responsiveness was assessed using the modified GRADE (Grading of Recommendations, Assessment, Development and Evaluation) approach [23]. This approach enables the quality of evidence for each property to be graded, starting at a high level of certainty and potentially downgraded to moderate, low or very low certainty based on four factors: (1) risk of bias (reflected in the quality appraisal according to the COSMIN criteria), (2) inconsistency (substantial unexplained heterogeneity with *I* ^2^ > 50%), (3) imprecision (sample size of the pooled estimate < 100) and (4) indirectness (evidence from populations, interventions or outcomes that differ from those relevant to this review) [23].

### 2.5 Data Analysis

A meta‐analysis was conducted to assess the psychometric properties of reliability, validity and responsiveness in studies using SarQoL, and a random effects model was applied given the expected heterogeneity between the studies (e.g., diagnosis of sarcopenia, characteristics of the population). A two‐sided *p*‐value of 0.05 or less was considered statistically significant for all findings, except for heterogeneity, which was considered significant if the *p*‐value was less than 0.1 [3]. R version 4.4.0 was used for all statistical analyses with the packages meta and tidyverse [24, 25]. Psychometric properties, which were reported in only one study, were reported in narrative form.

For the purpose of this study, the internal consistency results of one study, expressed as below a certain level, were considered as the level defined to be calculated in the meta‐analysis (e.g., *r* < 0.81 was considered as *r* = 0.81). In addition, when results were reported for the total population of a study first and the sarcopenic population specifically, then, the latter was considered before the total population.

To pool the estimates of the intraclass correlation coefficient (ICC), Cronbach's alpha and the correlation coefficients for reliability and construct validity, we applied Fisher's Z‐transformation, using an approximate variance determined by the sample size (z = 0.5 × ln((1 + *r*)/(1 − *r*)); Var(z) = 1/(*N* − 3)).

Heterogeneity was assessed using Cochran's Q test and Tau2 was used to measure between‐study variance. The *I* ^2^ statistic was used to quantify heterogeneity, with values of 25%, 50% and 75% representing low, moderate and substantial heterogeneity, respectively [26]. In case of significant heterogeneity, subgroup analyses were performed according to sarcopenia diagnosis, age of participants (> 75 years or < 75 years), continent, study population (total or sarcopenic population), RoB assessment, type of correlation (Spearman's or Pearson's) and in the specific case of test–retest reliability, stability of health status and time interval between the two administrations of the questionnaire. In addition, leave‐one‐out analyses were performed to assess the robustness of the results by removing one study at a time [27]. Asymmetry for publication bias was assessed using funnel plots and Egger's regression asymmetry test [28]. In case of publication bias, the Trim and Fill method was applied to evaluate the influence of potential missing studies on the pooled effect size [29].

## 3 Results

### 3.1 Study Selection

**FIGURE 1:**
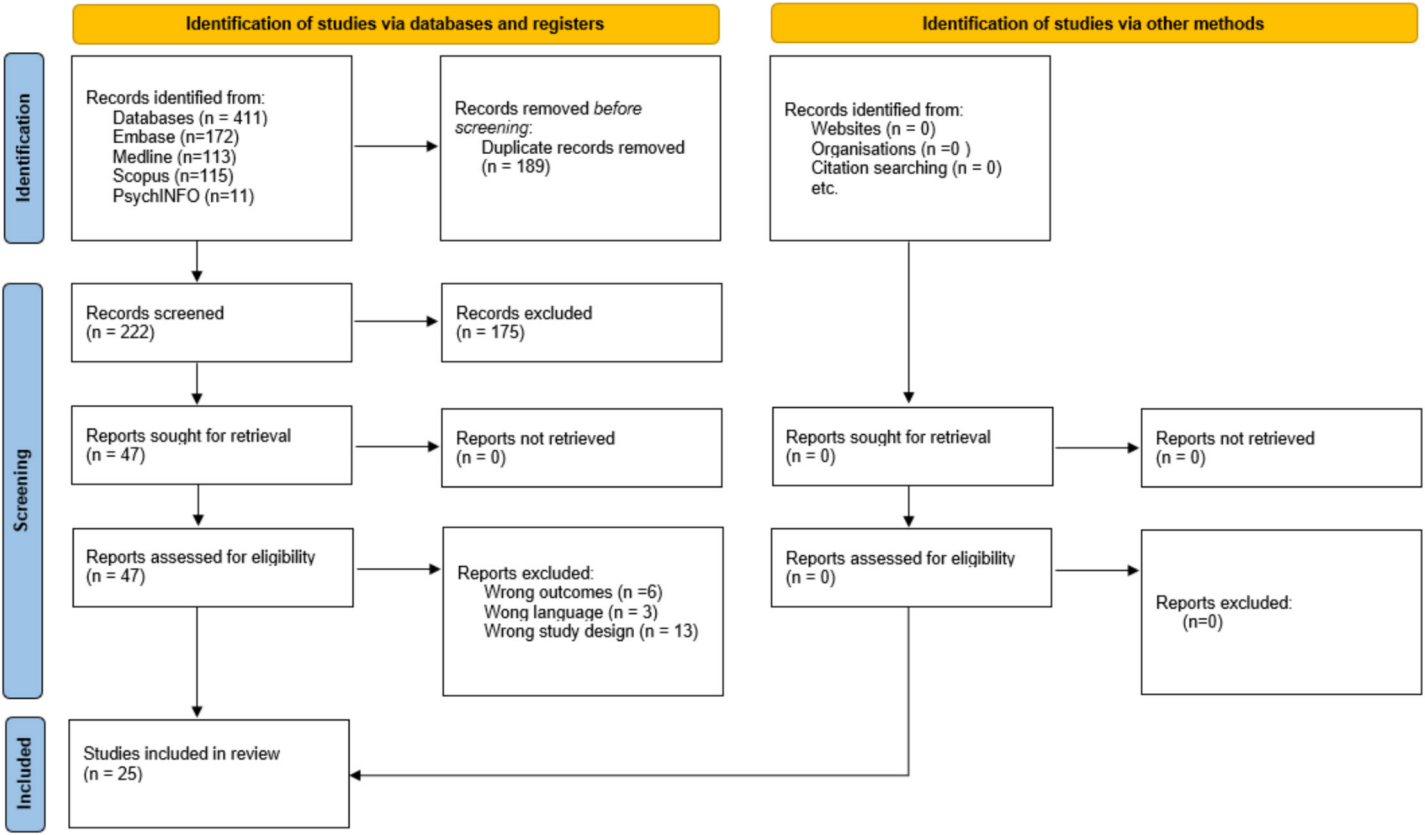
Flowchart of the identification and selection of studies.

The systematic electronic searches identified 411 potentially eligible studies. After removing duplicates, 222 references were initially screened for titles and abstracts, and 47 of these were screened based on their full text. Finally, 25 references met our inclusion criteria and were included in this systematic review (Figure 1).

### 3.2 Study Characteristics

The articles included were published between 2017 and 2024, and the data were combined from 4585 patients, of which 1311 were sarcopenic. Most studies were conducted in Europe (64%) and used the EWGSOP criteria to diagnose sarcopenia (76%). The characteristics of all included studies are reported in Table 2.

**TABLE 2:** Study characteristics.

First author's name, year	Continent	Sarcopenia definition (tools used to assess sarcopenia)	Participants (sample size, age, sex ratio)	Number of participants with sarcopenia (%)	Type of population	Psychometric properties measured
Alekna, 2019 [26]	Europe	EWGSOP2 Muscle strength: handgrip strength Lean mass: DXA Physical performance: SPPB	Sample size: 176, Age: 78.2 (74.1–82.6), Women: 59.7%	58 (33%)	Community dwelling older adults	Internal consistency Construct validity Test–retest reliability Floor and ceiling effect
Ariane, 2024 [30]	Asia	AWGS Muscle strength: grip strength Lean mass: BIA Physical performance: 6‐m walk test	Sample size: 176, Age: NR, Women: 61.0%	29 (49.1%)	Community dwelling older adults	Internal consistency Construct validity Test–retest reliability Floor and ceiling effect
Beaudart, 2017 [8]	Europe	EWGSOP1: Muscle strength: handgrip strength Lean mass: DXA Physical performance: SPPB	Sample size: 296, Age: 73.3 (68.9–78.6), Women: 57.1%	43 (14.5%)	Community dwelling older adults	Internal consistency Construct validity Test–retest reliability Floor and ceiling effect
Beaudart, 2017 [27]	Europe	EWGSOP1: Muscle strength: handgrip strength Lean mass: DXA Physical performance: gait speed	Sample size: 297, Age: 79.5 ± 2.62, Women: 46.1%	14 (4.7%) *Proportion of patients with low muscle function*	Community dwelling older adults	Internal consistency Construct validity Test–retest reliability Floor and ceiling effect
Demonceau, 2024 [31]	Europe	EWGSOP2: Muscle strength: handgrip strength Lean mass: DXA Physical performance: SPPB	Sample size: 17, Age: 82 ± 6.4, Women: 75.4%, + 11 Experts	17 (100%)	Community dwelling older adults Experts: 1 gerontologist, 4 geriatricians, 1 intensive care physician, 1 cardiologist	Content validity
de Souza Orlandi, 2023 [28]	South America	EWGSOP2: Muscle strength: handgrip strength Lean mass: DXA Physical performance: gait speed	Sample size: 221, Age: NR, Women: 68.3%	55 (25%)	Community dwelling older adults	Internal consistency Construct validity Test–retest reliability Floor and ceiling effect
Erdogan, 2021 [29]	Europe^a^	EWGSOP2: Muscle strength: handgrip strength Lean mass: BIA Physical performance: gait speed	Sample size: 100, Age: 74.7 ± 6.1, Women: 71%	5 (5%) *Proportion of sarcopenic patients with modified cut‐off*	Community dwelling older adults	Internal consistency Construct validity Test–retest reliability Floor and ceiling effect
Fabrega‐Cuadros, 2020 [32]	Europe	EWGSOP2: Muscle strength: handgrip strength Lean mass: BIA Physical performance: not assessed	Sample size: 252, Age: 74.00 (70.0–78.0), Women: 82.54%	66 (26%)	Community dwelling older adults	Internal consistency Construct validity Test–retest reliability Floor and ceiling effect
Gasparik, 2017 [33]	Europe	EWGSOP1 Muscle strength: handgrip strength Muscle mass: Lee equation Physical performance: gait speed	Sample size: 100, Age: 72 (67–79), Women: 69%	13 (13%) *Proportion of sarcopenic patients with modified cut‐off*	NR	Internal consistency Construct validity Floor and ceiling effect
Geerinck, 2018 [34]	Europe	EWGSOP1: Muscle strength: handgrip strength Lean mass: DXA Physical performance: SPPB	Sample size: 42, Age: 72.90 (68.85–78.81), Women: 59.5%	42 (100%)	Community dwelling older adults	Responsiveness
Geerinck, 2018 [35]	Europe	EWGSOP1: Muscle strength: handgrip strength Lean mass: BIA Physical performance: gait speed	Sample size: 92, Age: 82 (73–85), Women: 43.5%	30 (32.6%)	Community dwelling older adults	Internal consistency Construct validity Test–retest reliability Floor and ceiling effect
Geerinck, 2019 [36]	Multicentre (Belgium, Lithuania, UK, Brazil, Poland, Spain, Czech Republic, Greece)	EWGSOP/FNIH: Muscle strength: handgrip strength Lean mass: Lee equation–BIA–DXA Physical performance: gait speed–SPPB	Sample size: 278, Age: 77.67 ± 7.64, Women: 61.5%	278 (100%)	Community dwelling older adults	Measurement error
Geerinck, 2022 [37]	Europe	EWGSOP2: Muscle strength: handgrip strength Lean mass: BIA Physical performance: gait speed–5‐STS	Sample size: 70, Age: 80 (68.5–82.5), Women: 77.1%	30 (43%) *participants with low grip strength*	Community dwelling older adults, nursing home residents	Internal consistency Construct validity Floor and ceiling effect
Konstantynowicz, 2018 [38]	Europe	EWGSOP1: Muscle strength: handgrip strength Muscle mass: Lee equation Physical performance: /	Sample size: 106, Age: 73.3 ± 5.94, Women: 65.1%	60 (56.6%)	Community dwelling older adults	Internal consistency Construct validity Test–retest reliability Floor and ceiling effect
Kumar, 2023 [39]	Asia	AWGS: Muscle strength: handgrip strength Lean mass: BIA Physical performance: 5‐STS	Sample size: 114, Age: NR, Women: 40.3%	45 (39.5%)	Community dwelling older adults	Internal consistency Test–retest reliability Floor and ceiling effect
Le, 2021 [40]	Asia	AWGS Muscle strength: handgrip strength Muscle mass: Lee equation Physical performance: gait speed	Sample size: 159, Age: NR, Women: 46.5%	51 (32%)	Community dwelling older adults	Internal consistency Construct validity Test–retest reliability Floor and ceiling effect
Lee, 2023 [41]	Asia	AWGS: Muscle strength: handgrip strength Lean mass: BIA Physical performance: FTSTS–6MWT–SPPB	Sample size: 100, Age: 65–74: 48 (48%); 75–84: 37 (37%); 85+: 15 (15%), Women: 72 (72%)	50 (50%)	Community dwelling older adults	Internal consistency Construct validity Test–retest reliability Floor and ceiling effect
Mahmoodi, 2023 [42]	Asia	AWGS: Muscle strength: handgrip strength Lean mass: BIA Physical performance: Gait speed	Sample size: 128, Age: 74.78 ± 5.05, Women: 41.4%	88 (69%)	Community dwelling older adults	Internal consistency Construct validity Test–retest reliability Floor and ceiling effect
Martini, 2024 [43]	Europe	EWGSOP2: Muscle strength: grip strength Lean mass: DXA Physical performance: NR	Sample size: 185, Age: 79.8 ± 6.1, Women: 76.2%	51 (27.7%)	Community dwelling older adults	Internal consistency Construct validity Test–retest reliability Floor and ceiling effect
Matijević, 2020 [44]	Europe	EWGSOP2: Muscle strength: handgrip strength Lean mass: DXA Physical performance: gait speed	Sample size: 699, Age: 70 (67–74), Women: 72.7%	12 (2%)	Community dwelling older adults	Internal consistency Construct validity Floor and ceiling effect
Montero‐Errasquín, 2022 [45]	Europe	EWGSOP1–FNIH: Muscle strength: handgrip strength Lean mass: DXA Physical performance: SPPB *(only for EWGSOP1 definition)*	Sample size: 86, Age: 77.6 ± 5.3 (70–91), Women: 80.2%	16 (18.5%) *according to EWGSOP definition* 13 (15.1%) *according to FNIH definition*	Community dwelling older adults (but 1 participant in nursing home)	Internal consistency Construct validity Test–retest reliability Floor and ceiling effect
Tsekoura, 2020 [46]	Europe	EWGSOP1: Muscle strength: handgrip strength Lean mass: BIA Physical performance: gait speed	Sample size: 176, Age: 71.19 ± 7.95, Women: 77.27%	50 (28.5%)	Community dwelling older adults	Internal consistency Construct validity Test–retest reliability Floor and ceiling effect Responsiveness
Witham, 2022 [47]	Europe	EWGSOP2: Muscle strength: handgrip strength Lean mass: BIA Physical performance: SPPB	Sample size: 147, Age: 77.6 ± 7.3, Women: 49%	147 (100%)	Community dwelling older adults	Internal consistency Responsiveness
Yoo, 2021 [48]	Asia	EWGSOP2: Muscle strength: handgrip strength Lean mass: BIA Physical performance: /	Sample size: 450, Age: 73.9 ± 6.57, Women: 87.7%	53 (12%)	Community dwelling older adults	Internal consistency Construct validity Test–retest reliability Floor and ceiling effect
Yu, 2023 [49]	Asia	AWGS: Muscle strength: handgrip strength Lean mass: BIA Physical performance: gait speed	Sample size: 118, Age: NR, Women: 71.2%	58 (49%)	Community dwelling older adults	Internal consistency Construct validity Test–retest reliability Floor and ceiling effect

Abbreviations: 5‐STS/FTSTS: Five Times Sit To Stand Test; 6MWT: Six‐Minute Walk Test; AWGS: Asian Working Group on Sarcopenia; BIA: bioelectrical impedance analysis; DXA: dual energy X‐ray absorptiometry; EWGSOP: European Working Group on Sarcopenia in Older People; FNIH: Foundation for the National Institutes of Health Biomarkers Consortium Sarcopenia Project; NR: not reported; SPPB: Short Physical Performance Battery test.

^a^ Turkey was considered a European country because the Erdogan study used the EWGSOP2 definition of sarcopenia.

### 3.3 Quality Appraisal

The assessment of the quality of the psychometric properties obtained from the 25 included studies is presented in the Supporting Information (Table S1). Among the 22 studies that assessed ‘internal consistency’ and ‘hypothesis testing for construct validity’, all were rated as very good for these properties according to the COSMIN criteria [9, 30, 31, 32, 33, 34, 35, 36, 37, 38, 39, 40, 41, 42, 43, 45, 46, 47, 48, 49, 50, 51]. The quality of test–retest reliability varied between doubtful [31, 35, 36, 37, 38, 39], adequate [51] and very good [9, 30, 32, 33, 34, 40, 42, 43, 44, 45, 46, 47, 49] and the quality of the content validity was rated as inadequate in one study [47] and very good in the second [52] assessing this property.

### 3.4 Reliability

#### 3.4.1 Internal Consistency–Cronbach Alpha

**FIGURE 2:**
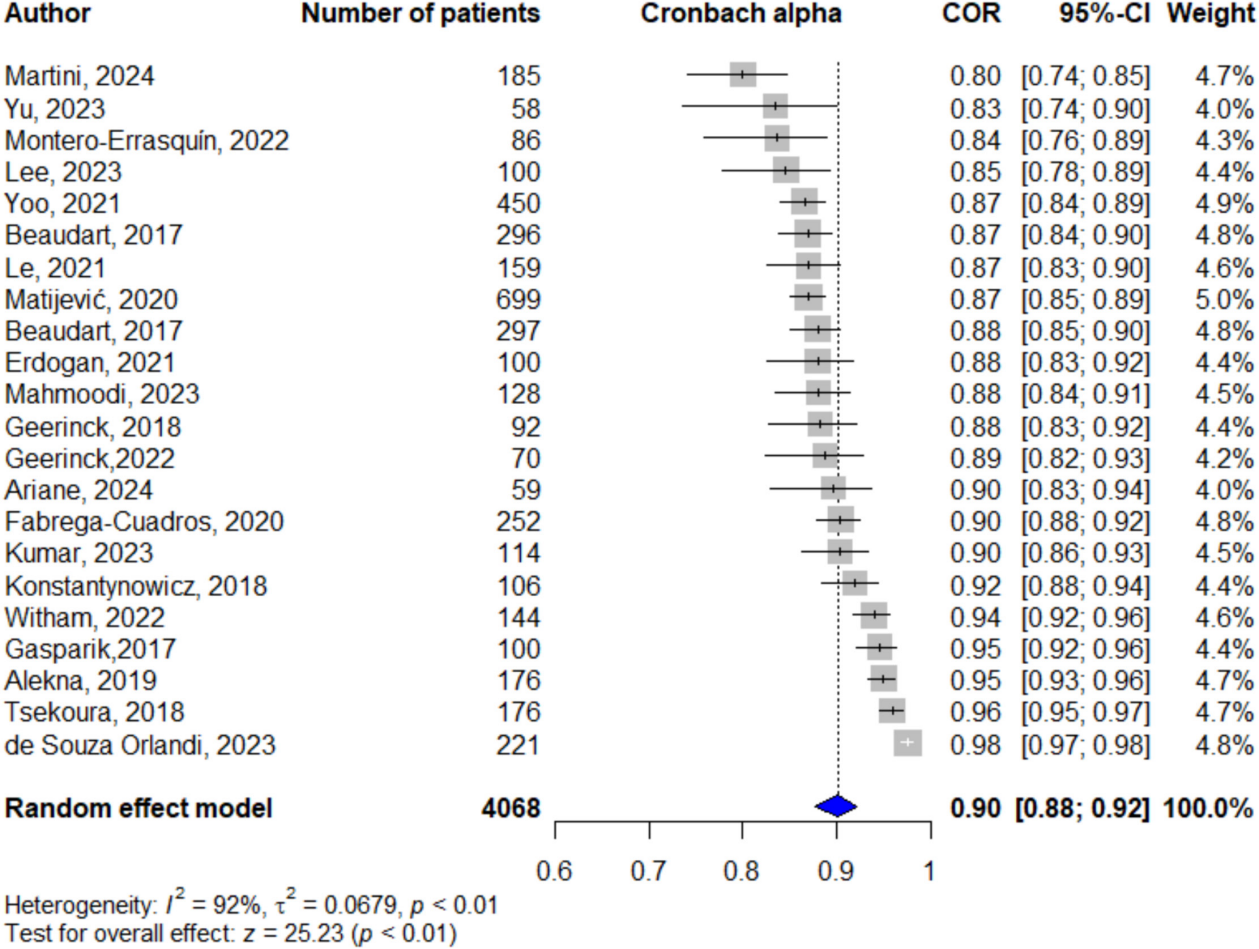
Forest plot of the internal consistency measured with Cronbach's alpha (global SarQoL score).

A total of 22 studies assessed the internal consistency of SarQoL global score [9, 30, 31, 32, 33, 34, 35, 36, 37, 38, 39, 40, 41, 42, 43, 45, 46, 47, 48, 49, 50, 51]. As shown in Figure 2, the pooled Cronbach's alpha was estimated at 0.90 (95% CI: 0.88; 0.92), demonstrating high reliability despite substantial heterogeneity (*I* ^2^ = 92%, Q‐test *p*‐value < 0.01).

As shown in Table 3, the number of studies assessing each SarQoL dimension ranged from 7 to 11, representing 773–1610 patients. For these individual dimensions, Cronbach's alpha ranged from 0.89 (95% CI: 0.85; 0.92) for dimension 2 ‘locomotion’ and dimension 4 ‘functionality’ to 0.82 (95% CI: 0.67; 0.90) for dimension 3 ‘body composition’, indicating high internal consistency. Significant heterogeneity was observed across all dimensions, with *I* ^2^ values ranging from 79% to 97% (Q‐test *p*‐values < 0.01).

**TABLE 3:** Meta‐analysis–internal consistency–Cronbach's alpha (random effect model).

	No. of studies	No. of patients	Cronbach's α (95% CI)	*I* ^2^	*p* for heterogeneity
Dimension 1	9	1213	0.85 (0.80; 0.89)	86%	< 0.0001
Dimension 2	9	1017	0.89 (0.85; 0.92)	89%	< 0.0001
Dimension 3	8	917	0.82 (0.67; 0.90)	97%	< 0.0001
Dimension 4	8	917	0.89 (0.85; 0.92)	79%	< 0.0001
Dimension 5	9	1214	0.87 (0.83; 0.90)	86%	< 0.0001
Dimension 6	11	1610	0.85 (0.75; 0.91)	97%	< 0.0001
Dimension 7	7	773	0.85 (0.73; 0.92)	95%	< 0.0001

*Note:* Dimension 1: physical and mental health; dimension 2: locomotion; dimension 3: body composition; dimension 4: functionality; dimension 5: activities of daily living; dimension 6: leisure activities; dimension 7: fears.

Abbreviation: CI: confidence interval.

Subgroup analyses (Table S2) showed that the geographic area where the study was performed influenced the internal consistency of the SarQoL global score. More specifically, in Asian countries, the pooled Cronbach's alpha of the global score was 0.87 (95% CI: 0.86; 0.89, *I* ^2^ = 0%) compared with 0.90 (95% CI: 0.88; 0.93, *I* ^2^ = 90%) in European countries (difference between subgroups *p* < 0.01). Subgroup analyses based on population type (total population versus sarcopenic population) and RoB assessment were not performed, as only one study reported Cronbach's alpha specifically in sarcopenic patients [31] and because the quality assessment of internal consistency was rated as ‘very good’ in all included studies.

The leave‐one‐out analysis did not identify any individual study as having a significant impact on the estimated effect size, and examination of the funnel plots and the results of Egger's tests did not reflect potential publication bias.

#### 3.4.2 Internal Consistency–Correlation Between Each Dimension and the SarQoL Global Score

A total of 21 studies assessed the correlation between the different dimensions and the global score of SarQoL [9, 30, 31, 32, 33, 34, 35, 36, 37, 38, 39, 41, 42, 43, 45, 46, 47, 48, 49, 50, 53], including between 3837 and 3907 patients. The pooled correlations, reported in Table 4, ranged from 0.47 (95% CI: 0.41; 0.54) for dimension 6 ‘leisure activities’ to 0.91 (95% CI: 0.89; 0.923) for dimension 4 ‘functionality’.

**TABLE 4:** Meta‐analysis–internal consistency–correlation between each dimension and SarQoL global score (random effect model).

	No. of studies	No. of patients	Correlation (95% CI)	*I* ^2^	*p* for heterogeneity
Dimension 1	20	3837	0.84 (0.82;0.86)	78%	< 0.0001
Dimension 2	20	3837	0.86 (0.83;0.89)	96%	< 0.0001
Dimension 3	20	3837	0.69 (0.63;0.73)	88%	< 0.0001
Dimension 4	21	3907	0.91 (0.89;0.93)	87%	< 0.0001
Dimension 5	20	3837	0.90 (0.87;0.92)	90%	< 0.0001
Dimension 6	20	3837	0.47 (0.41;0.54)	83%	< 0.0001
Dimension 7	21	3907	0.58 (0.51;0.63)	84%	< 0.0001

*Note:* Dimension 1: physical and mental health; dimension 2: locomotion; dimension 3: body composition; dimension 4: functionality; dimension 5: activities of daily living; dimension 6: leisure activities; dimension 7: fears.

Abbreviation: CI: confidence interval.

The models were associated with significant heterogeneity across all dimensions, with *I* ^2^ values ranging from 78% to 96% (Q‐test *p*‐values < 0.0001). No significant differences were observed in the subgroup analyses (Table S3). The leave‐one‐out analysis did not identify any individual study as having a significant impact on the estimated effect size, and examination of the funnel plots and the results of Egger's tests did not reflect potential publication bias for all dimensions.

#### 3.4.3 Test–Retest Reliability

Test–retest reliability, measured using the intraclass coefficient (ICC), was assessed in 17 studies for the global score, dimension 1 ‘physical and mental health’ and dimension 6 ‘leisure activities’, regrouping between 1087 and 1095 patients, and in 16 studies for the other dimensions, regrouping 1017 patients. The interval between the two test administrations was 3 days in 1 study, 2 weeks in 13 studies and not reported in 3 studies.

The pooled ICC was 0.98 (95% CI: 0.96; 0.99) for the global score, 0.94 (95% CI: 0.90; 0.97) for dimension 1 ‘physical and mental health’, 0.96 (95% CI: 0.93; 0.98) for dimension 2 ‘locomotion’, 0.93 (95% CI: 0.86; 0.97) for dimension 3 ‘body composition’, 0.97 (95% CI: 0.94; 0.98) for dimension 4 ‘functionality’, 0.96 (95% CI: 0.93; 0.97) for dimension 5 ‘activities of daily living’, 1 (95% CI: 1.00; 1.00) for dimensions 6 ‘leisure activities’ and 7 ‘fears’ (Table 5).

**TABLE 5:** Meta‐analysis–internal consistency–test–retest reliability (random effect model).

	No. of studies	No. of patients	ICC (95% CI)	*I* ^2^	*p* for heterogeneity
Global score	17	1087	0.98 (0.96;0.98)	93%	< 0.0001
Dimension 1	17	1095	0.94 (0.90;0.97)	96%	< 0.0001
Dimension 2	16	1017	0.96 (0.93;0.98)	95%	< 0.0001
Dimension 3	16	1017	0.93 (0.86;0.97)	97%	< 0.0001
Dimension 4	16	1017	0.97 (0.94;0.98)	93%	< 0.0001
Dimension 5	16	1017	0.96 (0.93;0.97)	94%	< 0.0001
Dimension 6	17	1095	1 (1.00;1.00)	88%	< 0.0001
Dimension 7	16	1017	1 (1.00;1.00)	93%	< 0.0001

*Note:* Dimension 1: physical and mental health; dimension 2: locomotion; dimension 3: body composition; dimension 4: functionality; dimension 5: activities of daily living; dimension 6: leisure activities; dimension 7: fears.

Abbreviations: CI: confidence interval, ICC: intraclass coefficient.

Subgroup analyses (Table S4) did not show a significant difference for the global SarQoL score. The leave‐one‐out analysis did not identify any individual study as having a significant impact on the estimated effect size, and the examination of the funnel plots and the results of Egger's tests did not reflect potential publication bias for the global score and all the dimensions of SarQoL.

#### 3.4.4 Measurement Error

Geerinck et al. reported the measurement error of SarQoL using the data from nine different cohorts, including 278 sarcopenic participants. First, they reported an SEM of 2.65 points, reflecting that there is 68% confidence that the true score of a sarcopenic patient is between −2.65 and +2.65 (out of 100) of the total SarQoL score obtained.

Secondly, by assessing the SDC, they highlighted that an individual change of at least 7.35 points out of 100 of the global SarQoL score needs to be observed to reflect that a true change has indeed occurred.

### 3.5 Validity

#### 3.5.1 Construct Validity

##### 3.5.1.1 Convergent Validity

Seventeen studies [9, 30, 31, 32, 33, 34, 35, 36, 37, 38, 40, 41, 45, 47, 48, 50] assessed the convergent validity of the SarQoL global score with at least one domain of the SF‐36 questionnaire. Six hypotheses were developed regarding the dimensions of the SF‐36 and confirmed the convergent validity with the SarQoL global score: bodily pain (k = 13, *r* = 0.53 [95% CI: 0.45; 0.61]), physical functioning (k = 16, *r* = 0.79 [95% CI: 0.72; 0.83]), limitation physical problem (k = 15, *r* = 0.60 [95% CI: 0.53; 0.66]), general health (k = 15, *r* = 0.58 [95% CI: 0.53; 0.63]), vitality (k = 17, *r* = 0.62 [95% CI: 0.53; 0.69]) and physical component score (k = 3, *r* = 0.75 [95% CI: 0.50; 0.89]) (Table 6).

**TABLE 6:** Meta‐analysis–validity–convergent/divergent validity.

	No. of studies	No. of patients	Correlation (95% CI)	*I* ^2^	*p* for heterogeneity
Convergent validity					
SF‐36					
Bodily pain	13	1320	0.53 (0.45; 0.61)	75%	< 0.01
Physical functioning	16	2373	0.79 (0.72; 0.83)	89%	< 0.01
Limitation physical problem	15	2085	0.60 (0.53; 0.66)	78%	< 0.01
General health	15	1676	0.58 (0.53; 0.63)	46%	0.02
Vitality	17	2434	0.62 (0.53; 0.69)	87%	< 0.01
Physical component score	3	294	0.75 (0.50; 0.89)	93%	< 0.01
Limitation emotional problem^a^	1	58	0.45 (0.21; 0.63)	—	—
Mental health^a^	1	58	0.62 (0.43; 0.46)	—	—
Mental component score^a^	1	106	0.62 (NR)	—	—
EQ‐5D					
Utility score	13	2356	0.61 (0.53; 0.68)	79%	< 0.01
Mobility	18	1939	−0.53 (−0.67; −0.36)	90%	< 0.01
Usual activity	18	1939	−0.51 (−0.65; −0.32)	91%	< 0.01
VAS	6	405	0.58 (0.41; 0.71)	79%	< 0.01
Pain/discomfort^a^	1	58	−0.47 (−0.65; −0.24)	—	—
Self‐care	2	79	0.04 (−0.88; 0.89)	96%	< 0.01
Anxiety/depression^a^	1	58	−0.62 (−0.76; −0.43)	—	—
Divergent validity					
SF‐36					
Mental health	11	1687	0.43 (0.21; 0.60)	94%	< 0.01
Social functioning	10	625	0.41 (0.24; 0.55)	79%	< 0.01
Limitation emotional problem	11	1324	0.40 (0.31; 0.48)	57%	0.009
Mental component score	2	188	0.30 (0.16; 0.42)	0%	0.83
Limitation physical problem^a^	1	50	0.41 (0.18–0.63)	—	—
EQ‐5D					
Self‐care	13	2016	−0.43 (−0.58; −0.25)	92%	< 0.01
Pain/discomfort	15	1644	−0.30 (−0.47; −0.11)	90%	< 0.01
Anxiety/depression	15	2046	−0.22 (−0.33; −0.10)	78%	< 0.01

Abbreviations: CI: confidence interval; EQ‐5D: EuroQol 5‐dimension; NR: not reported; SF‐36: 36‐Item Short Form Survey; VAS: visual analogic scale.

^a^ No meta‐analysis was performed because of the insufficient number of studies.

High level of heterogeneity was found across these six dimensions (*I* ^2^ between 46% and 93%).

Subgroup analyses (Table S5) showed that population type influenced the SarQoL global score for the SF‐36 ‘bodily pain’ dimension and age category for the SF‐36 ‘role physical’ dimension. The leave‐one‐out analysis did not identify any individual study substantially impacting the effect size. However, the omission of the study of Yoo et al. [36] contributed more to reducing heterogeneity. In fact, omitting this study reduced the *I* ^2^ for the dimension of ‘bodily pain’ from 75% to 28% and for the dimension ‘role physical’ from 79% to 56%. Examination of the funnel plot and the results of Egger's test for the ‘bodily pain’ dimension (*p* = 0.001) suggested potential publication bias. The Trim and Fill method was applied and identified 7 potential missing studies, which adjusted the pooled correlation to 0.64 (95% CI: 0.55; 0.72) instead of 0.54 (95% CI: 0.45; 0.61).

In addition, three other dimensions of the SF‐36 questionnaire were assessed quantitatively but not included in a meta‐analysis due to insufficient studies (k = 1). Yu et al. have reported positive correlations between SarQoL and ‘emotional role functioning’ (*r* = 0.45 [95% CI: 0.21; 0.64]) and ‘mental health’ (*r* = 0.62 [95% CI: 0.42; 0.76]) [31], and Konstantynowicz et al. reported a very good correlation of SarQoL with the ‘mental component score’ (*r* = 0.62 [*p* < 0.001]) [42].

Five dimensions of the EQ‐5D questionnaire were explored to test hypotheses of convergent validity and pooled in meta‐analyses showing positive and negative correlations with the global SarQoL score: utility score (k = 13, *r* = 0.61 [95% CI: 0.53; 0.68]), mobility (k = 18, *r* = −0.53 [95% CI: −0.67; −0.36]), usual activity (k = 18, *r* = −0.51 [95% CI: −0.65; −0.32]), visual analogic scale (k = 6, *r* = 0.58 [95% CI: 0.41; 0.71]) and self‐care (k = 2, *r* = 0.04 [95% CI: −0.88; 0.89]) (Table 6). Significant heterogeneity was observed for these dimensions (Table 6). Subgroup analyses (Table S5) did not detect significant differences between subgroups, except for the continent of the study for the EQ‐5D ‘usual activity’ dimension, where the pooled correlation was higher for studies conducted in Asia and Europe (−0.48 [95% CI: −0.74; −0.08], −0.49 [95% CI: −0.68; −0.25], respectively) compared to the study conducted in America (−0.72 [95% CI: −0.68; −0.65]). The leave‐one‐out analysis did not identify any individual study having a significant impact on the effect size.

In addition to these findings, Yoo et al. have reported ‘good’ and ‘very good’ correlations between the ‘pain and discomfort’ and ‘anxiety and depression’ EQ‐5D dimensions and the SarQoL global score. Erdogan et al. have also reported a negative correlation between the ‘self‐care’ dimension of the EQ‐5D and the SarQoL global score (*r* = −0.59, *p*‐value < 0.001).

##### 3.5.1.2 Divergent Validity

Four dimensions of the SF‐36 were explored to test the hypotheses of divergent validity and showed good correlations with the SarQoL global score (Table 6): mental health (k = 11, *r* = 0.43 [95% CI: 0.21; 0.60]), social functioning (k = 10, *r* = 0.41 [95% CI: 0.24; 0.55]), limitation of emotional problems (k = 11, *r* = 0.40 [95% CI: 0.31; 0.48]) and mental component score (k = 2, *r* = 0.30 [95% CI: 0.16; 0.42]). In addition, a good correlation of the SarQoL global score with the dimension of ‘limitation physical problem’ was reported by Tsekoura et al. (*r* = 0.41, *p* < 0.001) [30].

Finally, three dimensions of the EQ‐5D questionnaire were explored to test the hypotheses of divergent validity and showed a negative correlation with the total score of SarQoL: self‐care (k = 13, *r* = −0.43 [95% CI: −0.58; −0.25]), pain/discomfort (k = 15, *r* = −0.30 [95% CI: −0.47; −0.11]) and anxiety/depression (k = 15, *r* = −0.22 [95% CI: −0.33; −0.10]).

Significant heterogeneity was observed for all SF‐36 and EQ‐5D dimensions, except for the mental component score (*I* ^2^ = 0%, *p* = 0.83). Subgroup analyses (Table S5) showed a significant difference for the EQ‐5D ‘mental health’ dimension based on the continent of study (subgroup difference tests, *p* = 0.04). A higher correlation was observed for studies conducted in Europe (*r* = 0.55 [95% CI: 0.33; 0.72]) compared to studies conducted in Asia (*r* = 0.16 [95% CI: −0.17; 0.46]). The leave‐one‐out analysis showed that no study had a substantial impact on the overall effect sizes for divergent validity. In addition, examination of the funnel plots and Egger's tests showed no publication bias for the SF‐36 and EQ‐5D dimensions examined for divergent validity.

#### 3.5.2 Content Validity

Two studies have reported on the content validity of SarQoL [47, 52]. Mahmoodi et al. focused on the measured content validity of the tool based on expert opinion only and reported acceptable and appropriate content validity. However, the quality assessment of this study was rated as inadequate according to the COSMIN criteria due to the lack of assessment of comprehensiveness, comprehensibility and patient assessment. In contrast, Demonceau et al. assessed content validity with patient and expert perspectives and found an adequate relevance, comprehensiveness and comprehensibility of SarQoL. The quality of this study was rated as very good according to the COSMIN criteria.

### 3.6 Responsiveness

Two studies have assessed the responsiveness of SarQoL. The first, conducted by Geerinck et al. in 42 sarcopenic patients, showed good responsiveness to the questionnaire, with over 75% of hypotheses confirmed. The magnitude of change, expressed as the standardized mean difference, was significantly higher for the SarQoL total score than for the SF‐36 and EQ‐5D [54]. In a second study, Witham et al. assessed responsiveness in 147 participants with probable sarcopenia. They reported a weak correlation between SarQoL scores at baseline and after 6 months of follow‐up (*r* = 0.27, *p* = 0.03). This study also highlighted that SarQoL is more sensitive to improvements than to deteriorations, with sample sizes of 25–100 needed to detect clinically significant changes of 0.074 points [43].

### 3.7 Floor and Ceiling Effect

Floor and ceiling effects were assessed in 20 studies [9, 30, 31, 32, 33, 34, 36, 37, 39, 40, 41, 42, 45, 46, 47, 48, 49, 50] and none of these effects were observed.

### 3.8 Certainty of Evidence

Using the GRADE assessment, the certainty of evidence for reliability, validity and responsiveness was rated as high. Although substantial heterogeneity was observed for some psychometric properties, all effect estimates fell within the same interpretative range and pointed in the same direction. According to GRADE guidance, such variability, in the absence of opposing results or clinically divergent conclusions, does not justify downgrading for inconsistency. The observed heterogeneity is likely due to contextual differences (e.g., population or questionnaire application) rather than contradictory findings, as supported by the consistent direction of results across all studies [55].

## 4 Discussion

This systematic review and meta‐analysis aimed to report the psychometric properties of SarQoL, including reliability, validity, responsiveness and the presence of floor and ceiling effects. A total of 25 published studies were included, providing a comprehensive synthesis of its performance across different populations and settings.

The strong reliability of SarQoL was first demonstrated by robust internal consistency measured by Cronbach's alpha and Spearman's/Pearson's correlations. The pooled Cronbach's alpha values, which assess item homogeneity, were consistently above 0.80 for the global and dimension scores, exceeding the threshold of 0.70 to be considered adequate [16]. Subgroup analyses suggested that the SarQoL questionnaire may be slightly more tailored to European populations, as reflected by the higher pooled Cronbach's alpha observed in studies conducted in Europe. However, the excellent internal consistency (and absence of heterogeneity, probably due to a lower number of studies included in the model) demonstrated in the Asian continent also highlights the robust adaptability of the questionnaire across different cultural and geographical contexts, further supporting its applicability in international settings. Then, the correlations between the SarQoL global score and the individual dimensions (ranging from 0.48 to 0.91) further validated the consistency of the questionnaire. In addition, the test–retest reliability, reflected by the excellent ICC obtained of 0.98 for the global SarQoL score, underlined the ability of the questionnaire to provide stable results over time. Finally, the measurement error, reported in a single multicentre study [44], suggests that SarQoL is a reliable instrument. Indeed, this study highlighted that observed scores deviating from 2.65 points from the theoretical ‘true score’ and a smallest detectable change of 7.35 points were identified as the minimum change required to reflect a true difference in HRQoL in sarcopenic individuals. The authors have concluded that SarQoL has equivalent, if not superior, reliability to the SF‐36 questionnaire. Although only one study was included for this property, it consisted of 9 multicentre studies with a large and heterogeneous sample, giving good representativeness of measurement error, and we consider this sufficient to strengthen the other evidence for considering the reliability of SarQoL to be adequate.

The validity of SarQoL was first assessed using construct validity. Strong convergent correlations (*r* > 0.51) were observed with the SarQoL global score and related dimensions of the SF‐36 and EQ‐5D questionnaires, in addition to weaker correlations between the global SarQoL score and unrelated dimensions (*r* < 0.43) of the SF‐36 and EQ‐5D questionnaires, reflecting divergent validity. Interestingly, subgroup analyses for convergent validity showed significant differences for some dimensions, particularly according to type of population and age category. These differences were reflected in slightly lower correlations for individuals diagnosed with sarcopenia compared to the total population (individuals with and without sarcopenia) and higher correlations for people older than 75 years compared with those younger than 75 years. These differences may reflect that HRQoL in individuals with sarcopenia is perceived primarily in relation to physical and functional limitations, reducing the overlap between SarQoL and the more general SF‐36 questionnaire. On the other hand, they may also be due to the fact that older people experience more severe sarcopenia and more severe effects of sarcopenia, which are more in line with SarQoL and strengthen the correlations, while younger people, in better overall health, perceive less impact of sarcopenia on HRQoL, leading to weaker correlations [56]. Some other dimensions were investigated but not pooled in meta‐analysis because they were reported in only one study. These dimensions are difficult to interpret because, for example, they relate to convergent validity, although most studies used the same dimensions to assess divergent validity. In addition, one study reported that no correlation was found between SarQoL and some SF‐36 and EQ‐5D dimensions, such as ‘bodily pain’, ‘vitality’ and EQ‐VAS. This finding can be partly explained by the small sample size (*n* = 39) in this study [34]. Finally, an examination of the funnel plot and the results of Egger's test for the ‘bodily pain’ dimension (*p* = 0.001) suggested potential publication bias. This dimension of the SF‐36 questionnaire may be subject to publication bias because pain is often not considered as specific to sarcopenia compared with more specific dimensions related to functionality or mobility [57]. Variability in pain perception, comorbidities and population differences can also influence the results. However, the Trim and Fill method showed the inclusion of potentially missing studies, but did not significantly change the pooled estimate initially obtained and, therefore, did not affect the robustness of our results. Secondly, the validity of SarQoL was assessed through content validity. Since the creation of SarQoL, new guidelines for adequate content validity have been published, requiring an updated assessment [21]. In this context, two recent studies have specifically investigated this psychometric property. The study by Mahmoodi et al. concluded that the content validity of the questionnaire was adequate, but this study did not follow the updated COSMIN methodology, and we therefore rated the quality of this study as inadequate. Conversely, Demonceau et al. assessed the content validity and concluded that the questionnaire had adequate content validity with patients and experts. We rated this study as very good quality, as it met all the updated COSMIN criteria.

Although the responsiveness of SarQoL was only assessed in two studies, the results support an adequate level of responsiveness of which we are confident, particularly as the studies used two different methodological approaches. Nevertheless, it would be valuable for future research to further explore and strengthen the evidence for this psychometric property, especially in interventional studies. Moreover, the clinically relevant responsiveness threshold of SarQoL remains to be defined.

Finally, our analysis revealed the absence of floor and ceiling effects, indicating that SarQoL provides an adequate range of response options. This is an important finding, as it demonstrated the ability of the questionnaire to differentiate well between individuals with different levels of HRQoL. The absence of extreme values also improves its sensitivity and confirms the ability of SarQoL to accurately reflect the comprehensive spectrum of sarcopenia‐related HRQoL [21].

According to the GRADE approach, the certainty of evidence for reliability, validity and responsiveness was rated as ‘high.’ This high level of confidence is mainly justified by the fact that the unexplained heterogeneity does not affect the overall estimates or the direction of the results. In addition, the consistency of the findings across studies supports the robustness of the evidence, making a conservative approach to downgrading unnecessary in this context.

### 4.1 Strengths and Limitations

The key strength of this meta‐analysis lies in the fact that it is the first time that a quantitative approach has been used to assess the SarQoL psychometric properties, including a large sample of data from diverse geographic and cultural contexts, which underscores the wide applicability of SarQoL. Furthermore, by adhering to the COSMIN guidelines, this study ensures methodological rigor in evaluating the reliability, validity and responsiveness of SarQoL. Finally, the use of a meta‐analytic approach, including subgroup and sensitive analyses, reinforces the robustness of our findings.

However, this meta‐analysis is not without limitations. First, a large heterogeneity was observed across the various analyses, the causes of which remain largely unexplained. This could indicate inherent variations between the population or the application of the questionnaire. It is, however, important to consider that the statistical test *I* ^2^ may be subject to bias in small meta‐analyses, particularly with higher values when using a random effects model [58]. In addition, for some properties, such as internal consistency, although large heterogeneity was found, it could be argued that this does not affect the robustness of the results, as Cronbach's alpha remains above the recommended threshold with an extremely precise confidence interval. The observed heterogeneity can largely be attributed to the high precision of the studies, as the narrow 95% confidence intervals limit the chances of overlap. However, despite this heterogeneity, all results point in the same direction, demonstrating the consistency of the findings and strengthening the overall validity of the conclusions.

The majority of the included studies focus on community‐dwelling older adults with limited information on other specific patient settings. Further studies in different healthcare contexts, especially among individuals with severe sarcopenia or comorbidities, would help to strengthen our findings in these specific contexts, where the impact of sarcopenia on HRQoL may differ significantly.

Since multimorbidity is common in older individuals, another limitation of this study, also reported by Martinez‐Fernandez et al., is the potential impact of comorbidities on the patients' conditions and, more specifically, their functional capacity, which could influence their answers to the questionnaire [2, 11]. While these factors may affect the absolute scores obtained, they are not expected to impact the measurement properties of the questionnaire itself. This review focused on the evaluation of these properties, rather than on the interpretation of SarQoL scores as clinical outcomes.

## 5 Conclusion

The SarQoL questionnaire is the only disease‐specific PROM designed to assess HRQoL in people with sarcopenia. This meta‐analysis consolidates a decade of evidence and confirms the strong psychometric properties of SarQoL. The reliability, validity and responsiveness of SarQoL, supported by a high level of certainty according to the GRADE approach, reflect robust evidence. Furthermore, the absence of floor and ceiling effects underscores the ability of the questionnaire to capture a wide range of HRQoL variation, reinforcing its clinical utility.

## Funding

R.A.F. is partially supported by the US Department of Agriculture (USDA), under agreement No. 58‐8050‐3‐003, and by NIH Boston Claude D. Pepper Center (OAIC; 1P30AG031679).

## Conflicts of Interest

C.B., J.‐Y.R., O.B., I.B. and Y.R. are stakeholders of SARQOL SRL, a spin‐off of the University of Liège in charge of the interests of SarQoL. However, they did not receive any financial compensation for this role. R.A.F. reports grant support from Lonza, Biophytis, National Institutes of Health and USDA, scientific advisory board membership for Biophytis, Amazentis, Inside Tracker, Rejuvenate Biomed and Aging in Motion and consultancies for Embion, Biophytis, Amazentis, Pfizer, Nestlé and Rejuvenate Biomed. Other authors declare no conflicts of interest in relation to this work.

## Supporting information


**Table S1:** Methodological quality assessment according to the COSMIN checklist.
**Table S2:** Subgroups analysis–internal consistency–Cronbach's α.
**Table S3:** Subgroups analysis–internal consistency–correlation between each dimension and the SarQoL global score.
**Table S4:** Subgroups analysis–test‐retest–intraclass coefficient.
**Table S5:** Subgroups analysis–validity–convergent validity SF‐36/5Q‐5D Subgroup analyses based on the quality appraisal of the studies was not performed as they all were rated as ‘very good’ for construct validity.
**Table S6:** Subgroups analysis–validity–divergent validity SF‐36/5Q‐5D. Subgroup analyses based on the quality appraisal of the studies was not performed as they all were rated as ‘very good’ for construct validity.

## Data Availability

Data are available on request.
