# Supplementary material for: Psychometric Properties of the SarQoL Questionnaire: A Systematic Review and Meta‐Analysis
Source: J Cachexia Sarcopenia Muscle. 2025 Nov 23;16(6):e70122. doi: 10.1002/jcsm.70122 (PMC12646114; doi:10.1002/jcsm.70122)
Supplement: Supplementary file 1 — Table S1: Methodological quality assessment according to the COSMIN checklist. Table S2: Subgroups analysis–internal consistency–Cronbach's α. Table S3: Subgroups analysis–internal consistency–correlation between each dimension and the SarQoL global score. Table S4: Subgroups analysis–test‐retest–intraclass coefficient. Table S5: Subgroups analysis–validity–convergent validity SF‐36/5Q‐5D Subgroup analyses based on the quality appraisal of the studies was not performed as they all were rated as ‘very good’ for construct validity. Table S6: Subgroups analysis–validity–divergent validity SF‐36/5Q‐5D. Subgroup analyses based on the quality appraisal of the studies was not performed as they all were rated as ‘very good’ for construct validity. [file JCSM-16-e70122-s001.docx]

Supplementary material

S.1. The new PRISMA-COSMIN for Outcome Measurement Instrument


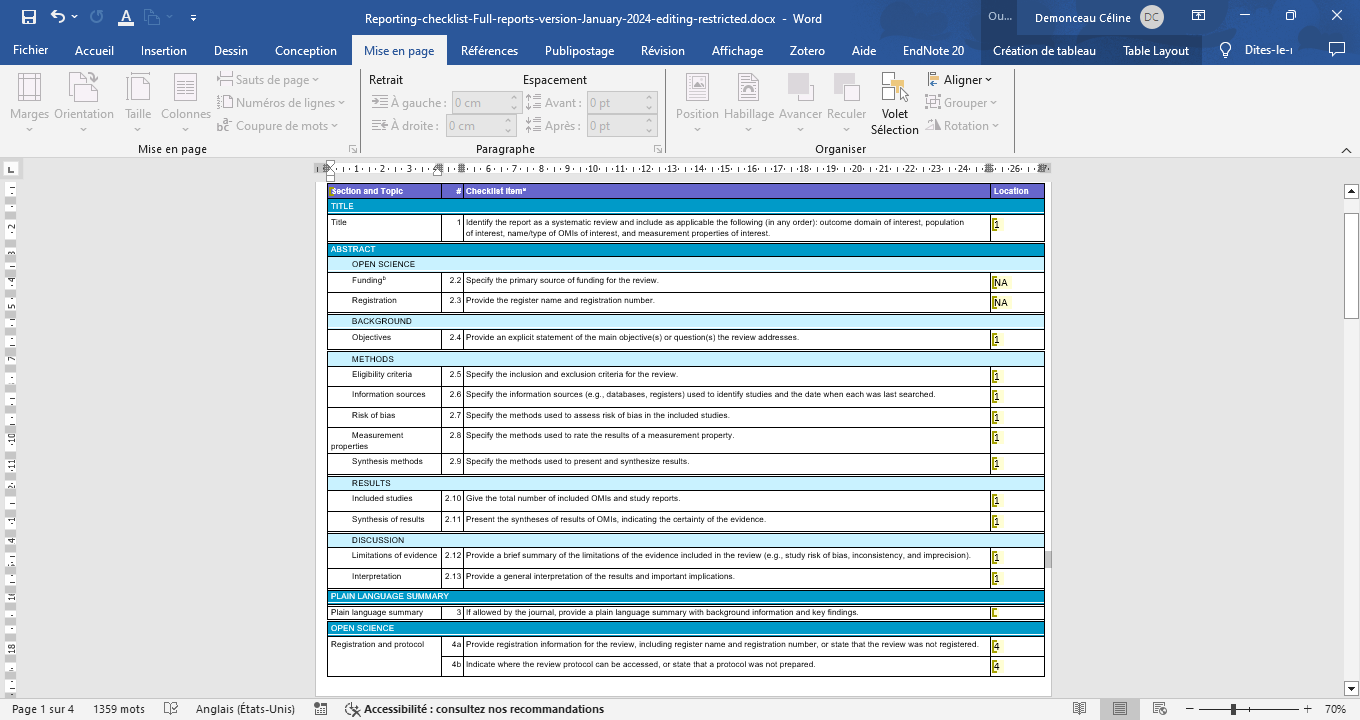


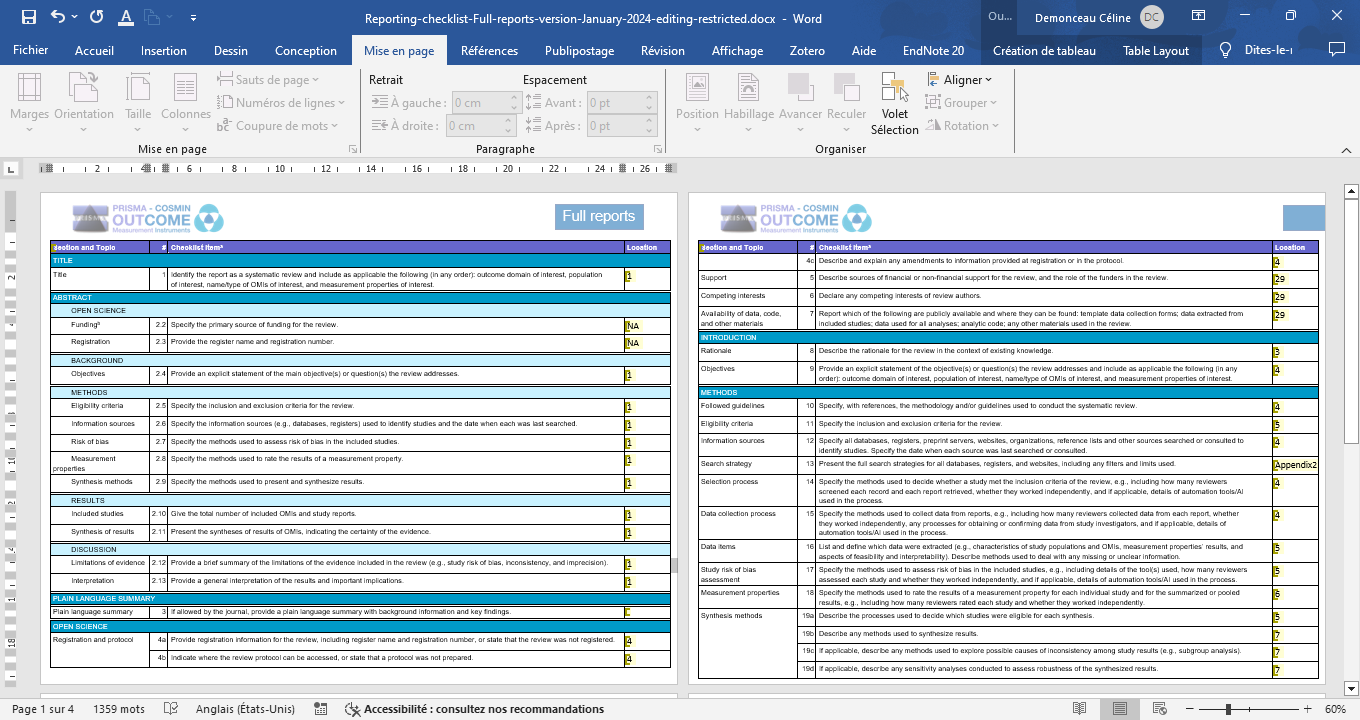


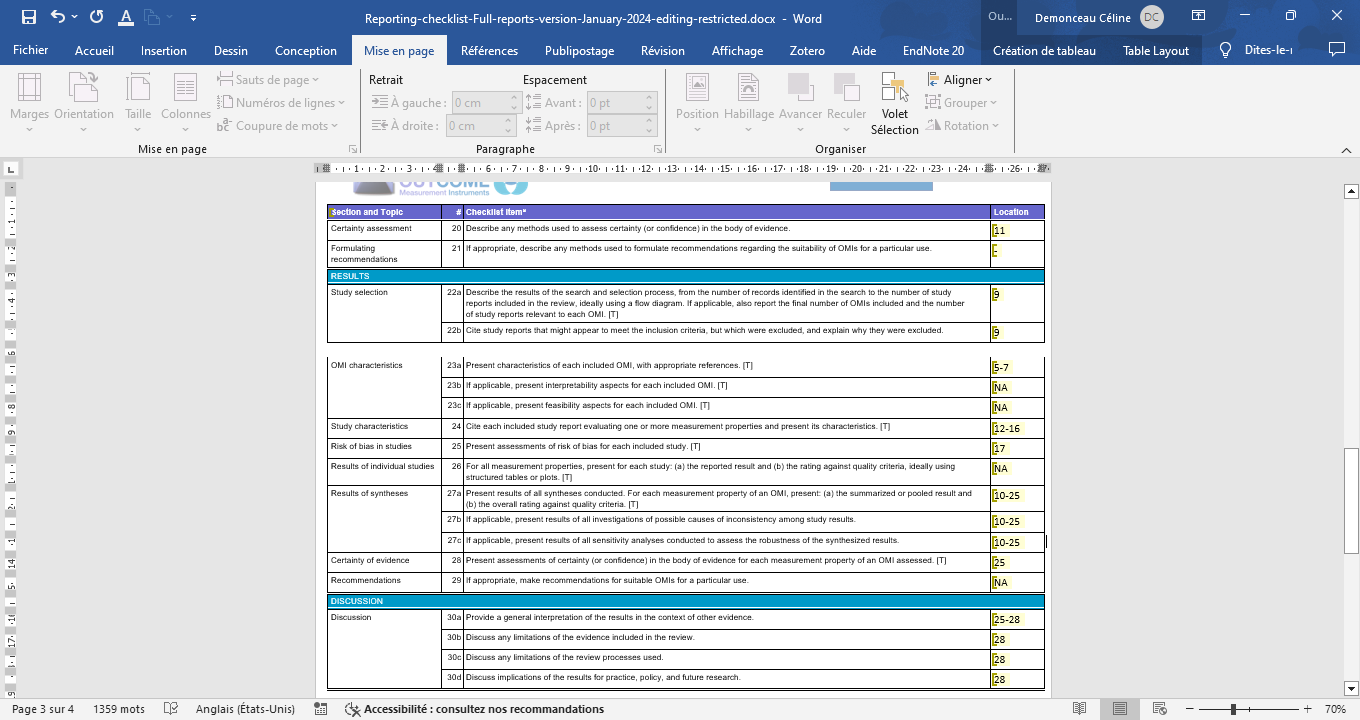


**S2. Search strategy (Medline via Ovid, Scopus, EMBASE and PsycINFO)**

Database: Ovid MEDLINE(R) ALL <1946 to February 27, 2024>
Search Strategy:

--------------------------------------------------------------------------------------

1 sarqol.ti,ab,kf.

2 "sarcopenia quality of life".ti,ab,kf.

3 "sarcopenia and quality of life".ti,ab,kf.

4 "sarcopenia & quality of life".ti,ab,kf.

5 or/1-4
*********

( TITLE-ABS-KEY ( sarqol ) ) OR ( TITLE-ABS-KEY
( "sarcopenia and quality of life" ) ) OR ( TITLE-ABS-KEY ( "sarcopenia quality of life" ) )
OR ( TITLE-ABS-KEY ( "sarcopenia & quality of life" ) )

EMBASE
.......................................................

#5. #1 OR #2 OR #3 OR #4 172 28 Feb 2024

#4. 'sarcopenia & quality of life':ti,ab 65 28 Feb 2024

#3. 'sarcopenia and quality of life':ti,ab 41 28 Feb 2024

#2. 'sarcopenia quality of life':ti,ab 65 28 Feb 2024

#1. sarqol:ti,ab,kw 136 28 Feb 2024

.......................................................

PSYCHINFO

Database: APA PsycInfo <1806 to February Week 4 2024>
Search Strategy:
---------------------------------------------------------------------------

1 sarqol.ti,ab.

2 "sarcopenia quality of life".ti,ab.

3 "sarcopenia and quality of life".ti,ab.

4 "sarcopenia & quality of life".ti,ab.

5 or/1-4

Table S1. **Methodological quality assessment according to the COSMIN checklist**

|  | | | | Content Validity | Internal consistency | | | Reliability  (test-retest) | | Hypotheses testing for construct validity | | Measurement error |
| --- | --- | --- | --- | --- | --- | --- | --- | --- | --- | --- | --- | --- |
| Alekna, 2019 [27] | | | | NR | V | | | V | | V | | NR |
| Ariane, 2024 [46] | | | | NR | V | | | V | | V | | NR |
| Beaudart, 2017 [8] | | | | NR | V | | | V | | V | | NR |
| Beaudart, 2017 [28] | | | | NR | V | | | V | | V | | NR |
| Demonceau, 2024 [49] | | | | V | NR | | | NR | | NR | | NR |
| de Souza Orlandi, 2023 [29] | | | | NR | V | | | D | | V | | NR |
| Erdogan, 2021 [30] | | | | NR | V | | | D | | V | | NR |
| Fabrega-Cuadros, 2020 [31] | | | | NR | V | | | D | | V | | NR |
| Gasparik, 2017 [32] | | | | NR | V | | | NR | | V | | NR |
| Geerinck, 2018 [50] | | | | NR | NR | | | NR | | NR | | V |
| Geerinck, 2018 [33] | | | | NR | V | | | V | | V | | NR |
| Geerinck, 2019 [48] | | | | NR | NR | | | V | | NR | | V |
| Geerinck, 2022 [34] | | | | NR | V | | | NR | | V | | NR |
| Konstantynowicz, 2018 [35] | | | | NR | V | | | V | | V | | NR |
| Kumar, 2023 [51] | | | | NR | V | | | A | | V | | NR |
| Le, 2021 [37] | | | | NR | V | | | V | | V | | NR |
| Lee, 2023 [38] | | | | NR | V | | | V | | V | | NR |
| Mahmoodi, 2023 [39] | | | | I | V | | | V | | V | | NR |
| Martini, 2024 [47] | | | | NR | V | | | D | | V | | NR |
| Matijević, 2020 [53] | | | | NR | V | | | NR | | V | | NR |
| Montero-Errasquin 2022 [41] | | | | NR | V | | | V | | V | | NR |
| Tsekoura, 2020 [42] | | | | NR | V | | | V | | V | | NR |
| Witham, 2022 [43] | | | | NR | V | | | V | | V | | NR |
| Yoo, 2021 [44] | | | | NR | V | | | D | | V | | NR |
| Yu, 2023 [45] | | | | NR | V | | | D | | V | | NR |
| V: very good; A: adequate; D: doubtful; I: inadequate; NR: not reported. | | | |  |  |  |  |  |  |  |  |  |
| **Table S2. Subgroups analysis – Internal consistency – Cronbach a** | | | |  |  |  |  |  |  |  |  |  |
|  | **No. studies** | **No. patients** | **Cronbach a (95% CI)** | | | **I^2^** | **P for heterogeneity** | | **P for subgroup differences** | |  |  |
| **Global SarQoL score** |  |  |  | | |  |  | |  | |  |  |
| Sarcopenia Diagnosis |  |  |  | | |  |  | | 0.38 | |  |  |
| *AWGS* | 6 | 618 | 0.88 (0.86; 0.89) | | | 6% |  | |  | |  |  |
| *EWGSOP1* | 8 | 1297 | 0.91 (0.88; 0.94) | | | 90% |  | |  | |  |  |
| *EWGSOP2* | 8 | 2153 | 0.91 (0.85; 0.94) | | | 96% | <0.0001 | |  | |  |  |
| Age category^1^ |  |  |  | | |  |  | | 0.49 | |  |  |
| *< 75 years* | 11 | 1798 | 0.92 (0.88; 0.94) | | | 94% | <0.0001 | |  | |  |  |
| *≥75 years* | 8 | 1816 | 0.90 (0.87; 0.92) | | | 87% | <0.0001 | |  | |  |  |
| Continent |  |  |  | | |  |  | | **<0.01** | |  |  |
| *Asia* | 7 | 1068 | 0.87 (0.86; 0.89) | | | 0% | 0.41 | |  | |  |  |
| *America* | 1 | 221 | 0.98 (0.97; 0.98) | | | / |  | |  | |  |  |
| *Europe* | 14 | 2779 | 0.90 (0.88; 0.93) | | | 90% | <0.0001 | |  | |  |  |
| CI: confidence interval, | | | |  |  |  |  |  |  |  |  |  |
| ^1^ 3 studies removed (Le, Lee, Ariane) because they did not report the age of total population of the study. | | | |  |  |  |  |  |  |  |  |  |

| **Table S3. Subgroups analysis – Internal consistency – Correlation between each dimension and the SarQoL global score.** | | | | | | | | | | | | | |
| --- | --- | --- | --- | --- | --- | --- | --- | --- | --- | --- | --- | --- | --- |
|  | **No. studies** | | | **No. patients** | | **Cronbach a (95% CI)** | **I^2^** | | **P for heterogeneity** | | | **P for subgroup differences** | |
| **Dimension 1** |  | | |  | |  |  | |  | | |  | |
| Sarcopenia Diagnosis |  | | |  | |  |  | |  | | | 0.67 | |
| *AWGS* | 6 | | | 549 | | 0.86 (0.81-0.89) | 66% | | 0.01 | | |  | |
| *EWGSOP1* | 7 | | | 1205 | | 0.83 (0.78-0.87) | 81% | | <0.01 | | |  | |
| *EWGSOP2* | 7 | | | 2083 | | 0.85 (0.81-0.88) | 80% | | <0.01 | | |  | |
| Population |  | | |  | |  |  | |  | | | 0.67 | |
| *Total population* | 18 | | | 3734 | | 0.85 (0.82-0.87) | 81% | | <0.01 | | |  | |
| *Sarcopenic population* | 2 | | | 103 | | 0.83 (0.76-0.88) | 0% | | 0.44 | | |  | |
| Age category ^1^ |  | | |  | |  |  | |  | | | 0.52 | |
| *< 75 years* | 9 | | | 2151 | | 0.83 (0.80-0.87) | 81% | | <0.01 | | |  | |
| *≥75 years* | 8 | | | 1368 | | 0.85 (0.81-0.88) | 76% | | <0.01 | | |  | |
| Continent |  | | |  | |  |  | |  | | | 0.05 | |
| *Asia* | 7 | | | 999 | | 0.86 (0.83-0.89) | 65% | | 0.02 | | |  | |
| *America* | 1 | | | 221 | | 0.79 (0.74-0.84) | / | |  | | |  | |
| *Europe* | 12 | | | 2617 | | 0.84 (0.81-0.87) | 81% | | <0.01 | | |  | |
| *Correlation type ^2^* |  | | |  | |  |  | |  | | | 0.71 | |
| *Spearman* | 8 | | | 1368 | | 0.85 (0.81-0.89) | 85% | | <0.01 | | |  | |
| *Pearson* | 7 | | | 1276 | | 0.84 (0.79-0.88) | 80% | | <0.01 | | |  | |
| *Methods* |  | | |  | |  |  | |  | | | 0.70 | |
| *Matrice* | 3 | | | 996 | | 0.85 (0.83-0.87) | 0% | | 0.52 | | |  | |
| *Per domain* | 17 | | | 2841 | | 0.84 (0.82-0.87) | 81% | | <0.01 | | |  | |
| **Dimension 2** |  | | |  | |  |  | |  | | |  | |
| Sarcopenia Diagnosis |  | | |  | |  |  | |  | | | 0.71 | |
| *AWGS* | 6 | | | 549 | | 0.88 (0.82-0.92) | 88% | | <0.01 | | |  | |
| *EWGSOP1* | 7 | | | 1205 | | 0.85 (0.78-0.90) | 92% | | <0.01 | | |  | |
| *EWGSOP2* | 7 | | | 2083 | | 0.86 (0.79-0.91) | 98% | | <0.01 | | |  | |
| Population |  | | |  | |  |  | |  | | | 0.38 | |
| *Total population* | 18 | | | 3734 | | 0.87 (0.83-0.90) | 96% | | <0.01 | | |  | |
| *Sarcopenic population* | 2 | | | 103 | | 0.84 (0.77-0.89) | 0% | | 0.51 | | |  | |
| Age category ^1^ |  | | |  | |  |  | |  | | | 0.42 | |
| *< 75 years* | 9 | | | 2151 | | 0.84 (0.76-0.89) | 97% | |  | | |  | |
| *≥75 years* | 8 | | | 1368 | | 0.87 (0.84-0.89) | 59% | |  | | |  | |
| Continent |  | | |  | |  |  | |  | | | 0.06 | |
| *Asia* | 7 | | | 999 | | 0.89 (0.83-0.92) | 86% | | <0.01 | | |  | |
| *America* | 1 | | | 221 | | 0.91 (0.88-0.93) | / | |  | | |  | |
| *Europe* | 12 | | | 2617 | | 0.85 (0.79-0.89) | 96% | | <0.01 | | |  | |
| *Correlation type ^2^* |  | | |  | |  |  | |  | | | 0.20 | |
| *Spearman* | 8 | | | 1368 | | 0.89 (0.86-0.92) | 85% | | <0.01 | | |  | |
| *Pearson* | 7 | | | 1276 | | 0.85 (0.78-0.90) | 92% | | <0.01 | | |  | |
| *Methods* |  | | |  | |  |  | |  | | |  | |
| *Matrice* | 3 | | | 996 | | 0.77 (0.55-0.90) | 98% | | <0.01 | | |  | |
| *Per domain* | 17 | | | 2841 | | 0.88 (0.85-0.90) | 89% | | <0.01 | | |  | |
| **Dimension 3** |  | | |  | |  |  | |  | | |  | |
| Sarcopenia Diagnosis |  | | |  | |  |  | |  | | | 0.31 | |
| *AWGS* | 6 | | | 549 | | 0.71 (0.60-0.79) | **82%** | | <0.01 | | |  | |
| *EWGSOP1* | 7 | | | 1206 | | 0.63 (0.53-0.71) | 82% | | <0.01 | | |  | |
| *EWGSOP2* | 7 | | | 2083 | | 0.72 (0.63-0.78) | 91% | | <0.01 | | |  | |
| Population |  | | |  | |  |  | |  | | | 0.17 | |
| *Total population* | 18 | | | 3734 | | 0.69 (0.64-0.74) | 89% | | <0.01 | | |  | |
| *Sarcopenic population* | 2 | | | 103 | | 0.60 (0.46-0.71) | 0% | | 0.75 | | |  | |
| Age category ^1^ |  | | |  | |  |  | |  | | | 0.49 | |
| *< 75 years* | 9 | | | 2151 | | 0.66 (0.56-0.74) | 92% | | <0.01 | | |  | |
| *≥75 years* | 8 | | | 1369 | | 0.70 (0.62-0.76) | 82% | | <0.01 | | |  | |
| Continent |  | | |  | |  |  | |  | | | 0.36 | |
| *Asia* | 7 | | | 999 | | 0.73 (0.63-0.80) | 85% | | <0.01 | | |  | |
| *America* | 1 | | | 221 | | 0.65 (0.56-0.72) | / | |  | | |  | |
| *Europe* | 12 | | | 2617 | | 0.66 (0.59-0.72) | 86% | | <0.01 | | |  | |
| *Correlation type ^2^* |  | | |  | |  |  | |  | | | 0.55 | |
| *Spearman* | 8 | | | 1368 | | 0.71 (0.61-0.78) | 90% | | <0.01 | | |  | |
| *Pearson* | 7 | | | 1276 | | 0.66 (0.54-0.76) | 93% | | <0.01 | | |  | |
| *Methods* |  | | |  | |  |  | |  | | | 0.96 | |
| *Matrice* | 3 | | | 996 | | 0.69 (0.65-0.72) | 22% | | 0.28 | | |  | |
| *Per domain* | 17 | | | 2841 | | 0.69 (0.63-0.74) | 90% | | <0.01 | | |  | |
| **Dimension 4** |  | | |  | |  |  | |  | | |  | |
| Sarcopenia Diagnosis |  | | |  | |  |  | |  | | | 0.07 | |
| *AWGS* | 6 | | | 549 | | 0.91 (0.87-0.94) | 83% | | <0.01 | | |  | |
| *EWGSOP1* | 7 | | | 1205 | | 0.88 (0.83-0.91) | 88% | | <0.01 | | |  | |
| *EWGSOP2* | 8 | | | 2153 | | 0.92 (0.91-0.94) | 84% | | <0.01 | | |  | |
| Population |  | | |  | |  |  | |  | | | 0.08 | |
| *Total population* | 19 | | | 3804 | | 0.91 (0.89-0.93) | 88% | | <0.01 | | |  | |
| *Sarcopenic population* | 2 | | | 103 | | 0.87 (0.81-0.91) | 0% | | 0.95 | | |  | |
| Age category ^1^ |  | | |  | |  |  | |  | | | 0.19 | |
| *< 75 years* | 9 | | | 2151 | | 0.89 (0.86-0.92) | 87% | | <0.01 | | |  | |
| *≥75 years* | 9 | | | 1438 | | 0.92 (0.89-0.94) | 85% | | <0.01 | | |  | |
| Continent |  | | |  | |  |  | |  | | | 0.73 | |
| *Asia* | 7 | | | 999 | | 0.91 (0.88-0.93) | 84% | | <0.01 | | |  | |
| *America* | 1 | | | 221 | | 0.92 (0.90-0.94) | / | |  | | |  | |
| *Europe* | 13 | | | 2687 | | 0.91 (0.88-0.93) | 90% | | <0.01 | | |  | |
| *Correlation type ^3^* |  | | |  | |  |  | |  | | | 0.06 | |
| *Spearman* | 8 | | | 1368 | | 0.92 (0.90-0.95) | 91% | | <0.01 | | |  | |
| *Pearson* | 7 | | | 1276 | | 0.88 (0.85-0.91) | 85% | | <0.01 | | |  | |
| *Methods* |  | | |  | |  |  | |  | | | 0.43 | |
| *Matrice* | 3 | | | 996 | | 0.92 (0.89-0.94) | 73% | | 0.02 | | |  | |
| *Per domain* | 18 | | | 2911 | | 0.91 (0.88-0.92) | 89% | | <0.01 | | |  | |
| **Dimension 5** |  | | |  | |  |  | |  | | |  | |
| Sarcopenia Diagnosis |  | | |  | |  |  | |  | | | 0.37 | |
| *AWGS* | 6 | | | 549 | | 0.89 (0.84-0.93) | 87% | | <0.01 | | |  | |
| *EWGSOP1* | 7 | | | 1205 | | 0.88 (0.82-0.93) | 94% | | <0.01 | | |  | |
| *EWGSOP2* | 7 | | | 2083 | | 0.92 (0.89-0.93) | 80% | | <0.01 | | |  | |
| Population |  | | |  | |  |  | |  | | | 0.17 | |
| *Total population* | 18 | | | 3734 | | 0.90 (0.88-0.92) | 91% | | <0.01 | | |  | |
| *Sarcopenic population* | 2 | | | 103 | | 0.87 (0.81-0.91) | 0% | |  | | |  | |
| Age category ^1^ |  | | |  | |  |  | |  | | | 0.63 | |
| *< 75 years* | 9 | | | 2151 | | 0.89 (0.85-0.92) | 91% | | <0.01 | | |  | |
| *≥75 years* | 8 | | | 1368 | | 0.90 (0.86-0.93) | 91% | | <0.01 | | |  | |
| Continent |  | | |  | |  |  | |  | | | 0.45 | |
| *Asia* | 7 | | | 999 | | 0.90 (0.86-0.93) | 86% | | <0.01 | | |  | |
| *America* | 1 | | | 221 | | 0.92 (0.89-0.94) | / | |  | | |  | |
| *Europe* | 12 | | | 2617 | | 0.90 (0.86-0.92) | 92% | | <0.01 | | |  | |
| *Correlation type ^2^* |  | | |  | |  |  | |  | | | 0.17 | |
| *Spearman* | 8 | | | 1368 | | 0.92 (0.90-0.94) | 82% | | <0.01 | | |  | |
| *Pearson* | 7 | | | 1276 | | 0.89 (0.82-0.93) | 94% | | <0.01 | | |  | |
| *Methods* |  | | |  | |  |  | |  | | | 0.61 | |
| *Matrice* | 3 | | | 996 | | 0.91 (0.89-0.92) | 36% | | 0.21 | | |  | |
| *Per domain* | 17 | | | 2841 | | 0.90 (0.87-0.92) | 91% | | <0.01 | | |  | |
| **Dimension 6** |  | | |  | |  |  | |  | | |  | |
| Sarcopenia Diagnosis |  | | |  | |  |  | |  | | | 0.86 | |
| *AWGS* | 6 | | | 549 | | 0.48 (0.33-0.60) | 79% | | <0.01 | | |  | |
| *EWGSOP1* | 7 | | | 1205 | | 0.50 (0.42-0.56) | 58% | | <0.01 | | |  | |
| *EWGSOP2* | 7 | | | 2083 | | 0.45 (0.30-0.58) | 91% | | <0.01 | | |  | |
| Population |  | | |  | |  |  | |  | | | 0.80 | |
| *Total population* | 18 | | | 3734 | | 0.48 (0.41-0.54) | 84% | | <0.01 | | |  | |
| *Sarcopenic population* | 2 | | | 103 | | 0.44 (0.09-0.69) | 72% | | 0.06 | | |  | |
| Age category ^1^ |  | | |  | |  |  | |  | | | 0.67 | |
| *< 75 years* | 9 | | | 2151 | | 0.44 (0.37-0.51) | 69% | | <0.01 | | |  | |
| *≥75 years* | 8 | | | 1368 | | 0.48 (0.33-0.60) | 90% | | <0.01 | | |  | |
| Continent |  | | |  | |  |  | |  | | | 0.50 | |
| *Asia* | 7 | | | 999 | | 0.45 (0.32-0.57) | 82% | | <0.01 | | |  | |
| *America* | 1 | | | 221 | | 0.54 (0.44-0.63) | / | |  | | |  | |
| *Europe* | 12 | | | 2617 | | 0.48 (0.39-0.56) | 84% | | <0.01; | | |  | |
| Correlation type ^2^ |  | | |  | |  |  | |  | | | 0.18 | |
| *Spearman* | 8 | | | 1368 | | 0.53 (0.40-0.64) | 89% | | <0.01 | | |  | |
| *Pearson* | 7 | | | 1276 | | 0.43 (0.35-0.51) | 68% | | <0.01 | | |  | |
| *Methods* |  | | |  | |  |  | |  | | | 0.68 | |
| *Matrice* | 3 | | | 996 | | 0.45 (0.28-0.59) | 81% | | <0.01 | | |  | |
| *Per domain* | 17 | | | 2841 | | 0.48 (0.40-0.55) | 84% | | <0.01 | | |  | |
| **Dimension 7** |  | | |  | |  |  | |  | | |  | |
| Sarcopenia Diagnosis |  | | |  | |  |  | |  | | | 0.91 | |
| *AWGS* | 6 | | | 549 | | 0.58 (0.50-0.65) | 65% | | <0.01 | | |  | |
| *EWGSOP1* | 7 | | | 1205 | | 0.56 (0.44-0.66) | 82% | | <0.01 | | |  | |
| *EWGSOP2* | 8 | | | 2153 | | 0.59 (0.47-0.70) | 91% | | <0.01 | | |  | |
| Population |  | | |  | |  |  | |  | | | 0.81 | |
| *Total population* | 19 | | | 3804 | | 0.58 (0.52-0.64) | 86% | | <0.01 | | |  | |
| *Sarcopenic population* | 2 | | | 103 | | 0.60 (0.45-0.71) | 0% | | 0.53 | | |  | |
| Age category ^1^ |  | | |  | |  |  | |  | | | 0.08 | |
| *< 75 years* | 9 | | | 2151 | | 0.62 (0.58-0.66) | 45% | | 0.10 | | |  | |
| *≥75 years* | 9 | | | 1438 | | 0.51 (0.36-0.63) | 89% | | <0.01 | | |  | |
| Continent |  | | |  | |  |  | |  | | | 0.89 | |
| *Asia* | 7 | | | 999 | | 0.59 (0.51-0.67) | 67% | | <0.01 | | |  | |
| *America* | 1 | | | 221 | | 0.60 (0.51-0.68) | / | |  | | |  | |
| *Europe* | 13 | | | 2687 | | 0.57 (0.47-0.65) | 89% | | <0.01 | | |  | |
| Correlation type ^3^ |  | | |  | |  |  | |  | | | 0.67 | |
| *Spearman* | 8 | | | 1368 | | 0.62 (0.50-0.71) | 90% | | <0.01 | | |  | |
| *Pearson* | 7 | | | 1276 | | 0.58 (0.47-0.68) | 83% | | <0.01 | | |  | |
| *Methods* |  | | |  | |  |  | |  | | | 0.56 | |
| *Matrice* | 3 | | | 996 | | 0.60 (0.56-0.64) | 0% | | 0.85 | | |  | |
| *Per domain* | 18 | | | 2911 | | 0.58 (0.50-0.64) | 87% | | <0.01 | | |  | |
| ^1^ 2 studies removed (Le, Lee) because they did not report the age of total population of the study.  ^2^ 4 studies removed (*Mahmoodi, Matijevic, Montero-Errasquin, de Souza Orlandi) because they did not report the type of correlation.*  ^3^ 5 studies removed (*Mahmoodi, Matijevic, Montero-Errasquin, de Souza Orlandi, Geerinck) because they did not report the type of correlation.*   \| **Table S4. Subgroups analysis – Test retest -Intraclass coefficient** \| \| \| \| \| \| \| \| --- \| --- \| --- \| --- \| --- \| --- \| --- \| \|  \| **No. studies** \| **No. patients** \| **ICC  (95% IC)** \| **I^2^** \| **P for heterogeneity** \| **P for subgroup differences** \| \| **Global score** \|  \|  \|  \|  \|  \|  \| \| Sarcopenia Diagnosis \|  \|  \|  \|  \|  \| 0.69 \| \| *AWGS* \| 6 \| 287 \| 0.98 (0.93; 0.99) \| 97% \| <0.0001 \|  \| \| *EWGSOP1* \| 5 \| 299 \| 0.97 (0.93; 0.98) \| 84% \| <0.0001 \|  \| \| *EWGSOP2* \| 6 \| 501 \| 0.98 (0.97; 0.98) \| 71% \| 0.004 \|  \| \| Population ^1^ \|  \|  \|  \|  \|  \| 0.47 \| \| *Total population* \| 3 \| 475 \| 0.96 (0.95; 0.97) \| 45% \| <0.0001 \|  \| \| *Sarcopenic population* \| 14 \| 622 \| 0.95 (0.90; 0.98) \| 97% \| <0.0001 \|  \| \| Age category ^2^ \|  \|  \|  \|  \|  \| 0.81 \| \| *< 75 years* \| 10 \| 789 \| 0.96 (0.90; 0.98) \| 97% \| <0.0001 \|  \| \| *≥ 75 years* \| 6 \| 269 \| 0.95 (0.91; 0.98) \| 86% \| <0.0001 \|  \| \| Continent \|  \|  \|  \|  \|  \| 0.19 \| \| *Asia* \| 7 \| 340 \| 0.98 (0.94; 0.99) \| 96% \| <0.0001 \|  \| \| *America* \| 1 \| 221 \| 0.98 (0.98; 0.99) \| - \| - \|  \| \| *Europe* \| 9 \| 526 \| 0.97 (0.96; 0.99) \| 81% \| <.0.0001 \|  \| \| *Quality of the study* \|  \|  \|  \|  \|  \| 0.62 \| \| *Very good* \| 10 \| 557 \| 0.98 (0.96; 0.99) \| 94% \| <0.0001 \|  \| \| *Adequate* \| 1 \| 25 \| 0.97 (0.93; 0.99) \| - \|  \|  \| \| *Doubtful* \| 6 \| 505 \| 0.97 (0.93; 0.99) \| 94% \| <0.0001 \|  \| \| *Time interval* \|  \|  \|  \|  \|  \| 0.14 \| \| *2-week interval* \| 13 \| 685 \| 0.98 (0.97; 0.99) \| 92% \| <0.0001 \|  \| \| *3-day interval* \| 1 \| 70 \| 0.96 (0.94; 0.98) \| - \| - \|  \| \| *Not specified* \| 3 \| 332 \| 0.96 (0.81; 0.99) \| 97% \| <0.0001 \|  \| \| *Patient stable* \|  \|  \|  \|  \|  \| 0.57 \| \| *Yes* \| 12 \| 695 \| 0.98 (0.96; 0.99) \| 93% \| <0.0001 \|  \| \| *Not specified* \| 5 \| 392 \| 0.97 (0.91; 0.99) \| 95% \| <0.0001 \|  \| \| *^1^* 1 study removed (Geerinck, 2019) because they did not report the population of the study *^2^* 2 studies removed (Ariane, Lee) because they did not report the age of the participants \| \| \| \| \| \| \| \|  \| \| \| \| \| \| \| | | | | | | | | | | | | | |
| **Table S5. Subgroups analysis – Validity – Convergent validity SF-36/5Q-5D** *Subgroup analyses based on the quality appraisal of the studies was not performed as they all were rated as ‘very good’ for construct validity* | | | | | | | | | | | | |  |
|  | | **No. studies** | **No. patients** | | **Cronbach a (95% CI)** | | | **I^2^** | | **P for heterogeneity** | **P for subgroup differences** | |  |
| **SF-36** | |  |  | |  | | |  | |  |  | |  |
| **Bodily pain** | |  |  | |  | | |  | |  |  | |  |
| Sarcopenia Diagnosis | |  |  | |  | | |  | |  | **0.56** | |  |
| *AWGS* | | 4 | 218 | | 0.46 (0.27; 0.61) | | | 54% | | 0.09 |  | |  |
| *EWGSOP1* | | 5 | 295 | | 0.54 (0.45; 0.62) | | | 0% | | 0.84 |  | |  |
| *EWGSOP2* | | 4 | 807 | | 0.58 (0.41; 0.71) | | | 87% | | <0.01 |  | |  |
| Population | |  |  | |  | | |  | |  | **0.03** | |  |
| *Total population* | | 5 | 920 | | 0.61 (0.51; 0.69) | | | 77% | | <0.01 |  | |  |
| *Sarcopenic population* | | 8 | 400 | | 0.46 (0.36; 0.56) | | | 26% | | 0.22 |  | |  |
| Age category | |  |  | |  | | |  | |  | 0.24 | |  |
| *< 75 years* | | 5 | 845 | | 0.60 (0.47; 0.70) | | | 81% | | <0.01 |  | |  |
| *≥75 years* | | 7 | 424 | | 0.51 (0.44; 0.58) | | | 0% | | 0.86 |  | |  |
| Continent | |  |  | |  | | |  | |  | 0.23 | |  |
| *Asia* | | 5 | 668 | | 0.54 (0.34; 0.69) | | | 87% | | <0.01 |  | |  |
| *America* | | 1 | 221 | | 0.61 (0.52; 0.68) | | | - | | - |  | |  |
| *Europe* | | 7 | 431 | | 0.51 (0.43; 0.58) | | | 0% | | 0.67 |  | |  |
| *Correlation type ^1^* | |  |  | |  | | |  | |  | 0.86 | |  |
| *Spearman* | | 6 | 967 | | 0.54 (0.39; 0.66) | | | 86% | | <0.01 |  | |  |
| *Pearson* | | 5 | 263 | | 0.53 (0.43; 0.61) | | | 0% | | 0.42 |  | |  |
| **Physical functioning** | |  |  | |  | | |  | |  |  | |  |
| Sarcopenia Diagnosis | |  |  | |  | | |  | |  | 0.79 | |  |
| *AWGS* | | 3 | 159 | | 0.80 (0.73; 0.85) | | | 0% | | 0.77 |  | |  |
| *EWGSOP1* | | 6 | 591 | | 0.80 (0.68; 0.89) | | | 94% | | <0.01 |  | |  |
| *EWGSOP2* | | 7 | 1623 | | 0.77 (0.67; 0.84) | | | 85% | | <0.01 |  | |  |
| Population | |  |  | |  | | |  | |  | 0.87 | |  |
| *Total population* | | 7 | 1915 | | 0.79 (0.70; 0.85) | | | 94% | | <0.01 |  | |  |
| *Sarcopenic population* | | 9 | 458 | | 0.78 (0.68; 0.85) | | | 80% | | <0.01 |  | |  |
| Age category | |  |  | |  | | |  | |  | 0.87 | |  |
| *< 75 years* | | 6 | 1775 | | 0.79 (0.68; 0.97) | | | 95% | | <0.01 |  | |  |
| *≥ 75 years* | | 10 | 598 | | 0.78 (0.70; 0.84) | | | 77% | | <0.01 |  | |  |
| Continent | |  |  | |  | | |  | |  | 0.08 | |  |
| *Asia* | | 4 | 609 | | 0.81 (0.77; 0.83) | | | 0% | | 0.76 |  | |  |
| *America* | | 1 | 221 | | 0.85 (0.81; 0.88) | | | - | | - |  | |  |
| *Europe* | | 11 | 1543 | | 0.77 (0.67; 0.84) | | | 91% | | <0.01 |  | |  |
| *Correlation type ^2^* | |  |  | |  | | |  | |  | 0.28 | |  |
| *Spearman* | | 9 | 1338 | | 0.76 (0.66; 0.84) | | | 93% | | <0.01 |  | |  |
| *Pearson* | | 4 | 246 | | 0.82 (0.75; 0.88) | | | 55% | | 0.09 |  | |  |
| **Role physical** | |  |  | |  | | |  | |  |  | |  |
| Sarcopenia Diagnosis | |  |  | |  | | |  | |  | 0.89 | |  |
| *AWGS* | | 4 | 217 | | 0.60 (0.38; 0.75) | | | 78% | | <0.01 |  | |  |
| *EWGSOP1* | | 4 | 245 | | 0.58 (0.48; 0.66) | | | 0% | | 0.65 |  | |  |
| *EWGSOP2* | | 7 | 221 | | 0.61 (0.50; 0.69) | | | 86% | | <0.01 |  | |  |
| Population | |  |  | |  | | |  | |  | 0.63 | |  |
| *Total population* | | 6 | 1619 | | 0.62 (0.52; 0.70) | | | 86% | | <0.01 |  | |  |
| *Sarcopenic population* | | 9 | 466 | | 0.58 (0.47; 0.68) | | | 63% | | <0.01 |  | |  |
| Age category | |  |  | |  | | |  | |  | **<0.01** | |  |
| *< 75 years* | | 5 | 1487 | | 0.67 (0.58; 0.74) | | | 85% | | <0.01 |  | |  |
| *≥ 75 years* | | 9 | 547 | | 0.51 (0.44; 0.58) | | | 6% | | 0.04 |  | |  |
| Continent | |  |  | |  | | |  | |  | 0.65 | |  |
| *Asia* | | 5 | 667 | | 0.64 (0.47; 0.77) | | | 87% | | <0.01 |  | |  |
| *America* | | 1 | 221 | | 0.56 (0.53; 0.67) | | | - | | - |  | |  |
| *Europe* | | 9 | 1197 | | 0.58 (0.52; 0.64) | | | 36% | | 0.13 |  | |  |
| *Correlation type ^2^* | |  |  | |  | | |  | |  | 0.47 | |  |
| *Spearman* | | 9 | 1134 | | 062 (0.53; 0.70) | | | 83% | | <0.01 |  | |  |
| *Pearson* | | 3 | 162 | | 0.54 (0.28; 0.72) | | | 71% | | 0.03 |  | |  |
| **General health** | |  |  | |  | | |  | |  |  | |  |
| Sarcopenia Diagnosis | |  |  | |  | | |  | |  | 0.37 | |  |
| *AWGS* | | 4 | 219 | | 0.62 (0.49; 0.73) | | | 41% | | 0.16 |  | |  |
| *EWGSOP1* | | 6 | 591 | | 0.60 (0.51; 0.68) | | | 48% | | 0.09 |  | |  |
| *EWGSOP2* | | 5 | 866 | | 0.54 (0.46; 0.61) | | | 47% | | 0.11 |  | |  |
| Population | |  |  | |  | | |  | |  | 0.84 | |  |
| *Total population* | | 6 | 1216 | | 0.57 (0.50; 0.64) | | | 61% | | 0.02 |  | |  |
| *Sarcopenic population* | | 9 | 460 | | 0.59 (0.50; 0.66) | | | 39% | | 0.11 |  | |  |
| Age category | |  |  | |  | | |  | |  | 0.21 | |  |
| *< 75 years* | | 6 | 1134 | | 0.61 (0.53; 0.68) | | | 64% | | 0.02 |  | |  |
| *≥ 75 years* | | 9 | 542 | | 0.54 (0.48; 0.61) | | | 5% | | 0.39 |  | |  |
| Continent | |  |  | |  | | |  | |  | 0.21 | |  |
| *Asia* | | 5 | 669 | | 0.61 (0.56; 0.66) | | | 23% | | 0.27 |  | |  |
| *America* | | 1 | 221 | | 0.52 (0.42; 0.61) | | | - | | - |  | |  |
| *Europe* | | 9 | 786 | | 0.56 (0.48; 0.64) | | | 56% | | 0.02 |  | |  |
| *Correlation type ^1^* | |  |  | |  | | |  | |  | 0.40 | |  |
| *Spearman* | | 7 | 1230 | | 0.57 (0.51; 0.63) | | | 53% | | 0.05 |  | |  |
| *Pearson* | | 6 | 356 | | 0.62 (0.52; 0.71) | | | 45% | | 0.10 |  | |  |
| **Vitality** | |  |  | |  | | |  | |  |  | |  |
| Sarcopenia Diagnosis | |  |  | |  | | |  | |  | 0.44 | |  |
| *AWGS* | | 4 | 220 | | 0.54 (0.30; 0.71) | | | 73% | | 0.01 |  | |  |
| *EWGSOP1* | | 6 | 591 | | 0.72 (0.58; 0.82) | | | 79% | | <0.01 |  | |  |
| *EWGSOP2* | | 7 | 1623 | | 0.55 (0.45; 0.64) | | | 90% | | <0.01 |  | |  |
| Population | |  |  | |  | | |  | |  | 0.91 | |  |
| *Total population* | | 7 | 1915 | | 0.61 (0.50; 0.70) | | | 93% | | <0.01 |  | |  |
| *Sarcopenic population* | | 10 | 519 | | 0.62 (0.47; 0.73) | | | 79% | | <0.01 |  | |  |
| Age category | |  |  | |  | | |  | |  | 0.90 | |  |
| *< 75 years* | | 7 | 1833 | | 0.61 (0.49; 0.71) | | | 93% | | <0.01 |  | |  |
| *≥ 75 years* | | 10 | 601 | | 0.62 (0.48; 0.73) | | | 79% | | <0.01 |  | |  |
| Continent | |  |  | |  | | |  | |  | 0.13 | |  |
| *Asia* | | 5 | 670 | | 0.49 (0.28; 0.65) | | | 83% | | <0.01 |  | |  |
| *America* | | 1 | 221 | | 0.57 (0.48; 0.65) | | | - | | - |  | |  |
| *Europe* | | 11 | 1543 | | 0.67 (0.57; 0.74) | | | 74% | | <0.01 |  | |  |
| *Correlation type ^2^* | |  |  | |  | | |  | |  | 0.40 | |  |
| *Spearman* | | 7 | 1230 | | 0.57 (0.51; 0.63) | | | 53% | | 0.05 |  | |  |
| *Pearson* | | 6 | 356 | | 0.62 (0.52; 0.71) | | | 45% | | 0.10 |  | |  |
| **Physical component score** | |  |  | |  | | |  | |  |  | |  |
| Sarcopenia Diagnosis | |  |  | |  | | |  | |  | **<0.01** | |  |
| *AWGS* | | 2 | 188 | | 0.65 (0.44; 0.80) | | | 78% | | 0.03 |  | |  |
| *EWGSOP1* | | 1 | 106 | | 0.88 (0.83; 0.92) | | | - | | - |  | |  |
| Population | |  |  | |  | | |  | |  | **0.03** | |  |
| *Total population* | | 2 | 206 | | 0.82 (0.62; 0.92) | | | 90% | | <0.01 |  | |  |
| *Sarcopenic population* | | 1 | 88 | | 0.55 (0.39; 0.68) | | | 90% | | <0.01 |  | |  |
| Age category ^3^ | |  |  | |  | | |  | |  | **<0.01** | |  |
| *< 75 years* | | 1 | 106 | | 0.88 (0.83; 0.92) | | | - | | - |  | |  |
| *≥ 75 years* | | 1 | 88 | | 0.55 (0.39: 0.68) | | | - | |  |  | |  |
| Continent | |  |  | |  | | |  | |  | **<0.01** | |  |
| *Asia* | | 2 | 188 | | 0.65 (0.44; 0.80) | | | 78% | | 0.03 |  | |  |
| *Europe* | | 1 | 106 | | 0.88 (0.83; 0.92) | | | - | | - |  | |  |
| *Correlation type* | |  |  | |  | | |  | |  | **0.03** | |  |
| *Spearman* | | 2 | 206 | | 0.82 (0.62; 0.92) | | | 90% | | <0.01 |  | |  |
| *Pearson* | | 1 | 88 | | 0.55 (0.39; 0.68) | | | - | | - |  | |  |
| **EQ-5D** | |  |  | |  | | |  | |  |  | |  |
| **Utility score** | |  |  | |  | | |  | |  |  | |  |
| Sarcopenia Diagnosis | |  |  | |  | | |  | |  | 0.36 | |  |
| *AWGS* | | 2 | 172 | | 0.60 (0.36; 0.77) | | | 74% | | <0.01 |  | |  |
| *EWGSOP1* | | 5 | 631 | | 0.67 (0.54; 0.77) | | | 86% | | <0.01 |  | |  |
| *EWGSOP2* | | 6 | 1553 | | 0.56 (0.48; 0.64) | | | 75% | | <0.01 |  | |  |
| Population | |  |  | |  | | |  | |  | 0.30 | |  |
| *Total population* | | 8 | 2065 | | 0.58 (0.49; 0.66) | | | 80% | | <0.01 |  | |  |
| *Sarcopenic population* | | 5 | 291 | | 0.66 (0.52; 0.77) | | | 73% | | <0.01 |  | |  |
| Age category | |  |  | |  | | |  | |  | 0.95 | |  |
| *< 75 years* | | 9 | 2053 | | 0.61 (0.52; 0.69) | | | 80% | | <0.01 |  | |  |
| *≥75 years* | | 4 | 303 | | 0.60 (0.43; 0.74) | | | 79% | | <0.01 |  | |  |
| Continent | |  |  | |  | | |  | |  | 0.15 | |  |
| *Asia* | | 3 | 622 | | 0.55 (0.39; 0.67) | | | 69% | | 0.04 |  | |  |
| *America* | | 1 | 221 | | 0.51 (0.41; 0.60) | | | 79% | | - |  | |  |
| *Europe* | | 9 | 1513 | | 0.64 (0.54; 0.72) | | | 80% | | <0.01 |  | |  |
| *Correlation type ^4^* | |  |  | |  | | |  | |  | 0.59 | |  |
| *Spearman* | | 8 | 1214 | | 0.61 (0.50; 0.70) | | | 84% | | <0.01 |  | |  |
| *Pearson* | | 3 | 222 | | 0.66 (0.47; 0.79) | | | 77% | | <0.01 |  | |  |
| **Mobility** | |  |  | |  | | |  | |  |  | |  |
| Sarcopenia Diagnosis | |  |  | |  | | |  | |  | 0.53 | |  |
| *AWGS* | | 6 | 431 | | -0.48 (-0.78; -0.02) | | | 90% | | 0.01 |  | |  |
| *EWGSOP1* | | 6 | 591 | | -0.46 (-0.73; -0.06) | | | 94% | | <0.01 |  | |  |
| *EWGSOP2* | | 6 | 917 | | -0.62 (-0.71; -0.51) | | | 85% | | <0.01 |  | |  |
| Population | |  |  | |  | | |  | |  | 0.48 | |  |
| *Total population* | | 8 | 1430 | | -0.58 (-0.68; -0.47) | | | 88% | | <0.01 |  | |  |
| *Sarcopenic population* | | 10 | 509 | | -0.48 (-0.76; -0.37) | | | 85% | | <0.01 |  | |  |
| Age category ^6^ | |  |  | |  | | |  | |  | 0.32 | |  |
| *< 75 years* | | 7 | 1248 | | -0.43 (-0.66; -0.13) | | | 93% | | <0.01 |  | |  |
| *≥75 years* | | 10 | 591 | | -0.60 (-0.74; -0.61) | | | 47% | | 0.06 |  | |  |
| Continent | |  |  | |  | | |  | |  | 0.12 | |  |
| *Asia* | | 7 | 881 | | -0.48 (-0.74; -0.11) | | | 90% | | <0.01 |  | |  |
| *America* | | 1 | 221 | | -0.70 (-0.76; -0.62) | | | - | | <0.01 |  | |  |
| *Europe* | | 10 | 837 | | -0.54 (-0.71; -0.32) | | | 91% | | <0.01 |  | |  |
| *Correlation type ^5^* | |  |  | |  | | |  | |  | 0.39 | |  |
| *Spearman* | | 12 | 1504 | | -0.60 (-0.67; -0.51) | | | 81% | | <0.01 |  | |  |
| *Pearson* | | 3 | 124 | | -0.30 (-0.82; 0.49) | | | 96% | | <0.01 |  | |  |
| **Usual activity** | |  |  | |  | | |  | |  |  | |  |
| Sarcopenia Diagnosis | |  |  | |  | | |  | |  | 0.80 | |  |
| *AWGS* | | 6 | 431 | | -0.48 (-0.78; 0.01) | | | 92% | | <0.01 |  | |  |
| *EWGSOP1* | | 6 | 591 | | -0.46 (-0.76; 0.00) | | | 94% | | <0.01 |  | |  |
| *EWGSOP2* | | 6 | 917 | | -0.57 (-0.67; -0.45) | | | 85% | | <0.01 |  | |  |
| Population | |  |  | |  | | |  | |  | 0.43 | |  |
| *Total population* | | 8 | 1430 | | -0.57 (-0.64; -0.50) | | | 77% | | <0.01 |  | |  |
| *Sarcopenic population* | | 10 | 509 | | -0.44 (-0.72; -0.05) | | | 94% | | <0.01 |  | |  |
| Age category^3^ | |  |  | |  | | |  | |  | 0.46 | |  |
| *< 75 years* | | 7 | 1248 | | -0.41 (-0.68; -0.05) | | | 95% | | <0.01 |  | |  |
| *≥75 years* | | 10 | 591 | | -0.56 (-0.74; -0.31) | | | 86% | | <0.01 |  | |  |
| Continent | |  |  | |  | | |  | |  | 0.03 | |  |
| *Asia* | | 7 | 881 | | -0.48 (-0.74; -0.08) | | | 91% | | <0.01 |  | |  |
| *America* | | 1 | 221 | | -0.72 (-0.78; -0.65) | | | - | | - |  | |  |
| *Europe* | | 10 | 837 | | -0.49 (-0.68; -0.25) | | | 90% | | <0.01 |  | |  |
| *Correlation type ^5^* | |  |  | |  | | |  | |  | 0.44 | |  |
| *Spearman* | | 12 | 1504 | | -0.59 (-0.66; -0.51) | | | 74% | | <0.01 |  | |  |
| *Pearson* | | 3 | 124 | | -0.26 (-0.86; 0.66) | | | 96% | | <0.01 |  | |  |
| **VAS** | |  |  | |  | | |  | |  |  | |  |
| Sarcopenia Diagnosis | |  |  | |  | | |  | |  | **<0.01** | |  |
| *AWGS* | | 1 | 20 | | 0.28 (-0.18; 0.65) | | | - | | - |  | |  |
| *EWGSOP1* | | 2 | 198 | | 0.74 (0.67; 0.81) | | | 16% | | 0.27 |  | |  |
| *EWGSOP2* | | 3 | 187 | | 0.48 (0.36; 0.59) | | | 0% | | 0.48 |  | |  |
| Population | |  |  | |  | | |  | |  | **0.01** | |  |
| *Total population* | | 3 | 268 | | 0.69 (0.55; 0.80) | | | 71% | | 0.03 |  | |  |
| *Sarcopenic population* | | 3 | 137 | | 0.42 (0.27; 0.55) | | | 0% | | 0.59 |  | |  |
| Age category | |  |  | |  | | |  | |  | 0.09 | |  |
| *< 75 years* | | 1 | 106 | | 0.71 (0.60; 0.79) | | | - | |  |  | |  |
| *≥75 years* | | 5 | 299 | | 0.54 (0.33; 0.70) | | | 80% | | <0.01 |  | |  |
| Continent | |  |  | |  | | |  | |  | 0.13 | |  |
| *Asian* | | 1 | 20 | | 0.28 (-0.18; 0.65) | | | - | | - |  | |  |
| *Europe* | | 5 | 385 | | 0.61 (0.44; 0.74) | | | 80% | | 0.01 |  | |  |
| *Correlation type ^5^* | |  |  | |  | | |  | |  | **0.02** | |  |
| *Spearman* | | 3 | 223 | | 0.55 (0.31; 0.72) | | | 80% | | <0.01 |  | |  |
| *Pearson* | | 1 | 92 | | 0.78 (0.68; 0.85) | | | - | | - |  | |  |
| ^1^ 1 study removed (Geerinck 2022) because they did not report the type of correlation used.  ^2^ 2 studies removed (Geerinck, *Matijevic)* because they did not report the type of correlation used. ^3^ 1 study removed (Lee) because they did not report the age of the total population  ^4^ 2 studies removed (Matijevic, de Souza Orlandi) because they did not report the type of correlation used  ^5^ 2 studies removed (Geerinck,2022, de Souza Orlandi) because they did not report the type of correlation used  ^6^ 1 study removed (Lee) because they did not report the age category   \| **Table S6. Subgroups analysis – Validity – Divergent validity SF-36/5Q-5D** *Subgroup analyses based on the quality appraisal of the studies was not performed as they all were rated as ‘very good’ for construct validity* \| \| \| \| \| \| \| \| --- \| --- \| --- \| --- \| --- \| --- \| --- \| \|  \| **No. studies** \| **No. patients** \| **Cronbach a (95% CI)** \| **I^2^** \| **P for heterogeneity** \| **P for subgroup differences** \| \| **SF-36** \|  \|  \|  \|  \|  \|  \| \| **Mental health** \|  \|  \|  \|  \|  \|  \| \| Sarcopenia Diagnosis \|  \|  \|  \|  \|  \| 0.13 \| \| *AWGS* \| 3 \| 159 \| 0.25 (-0.15; 0.58) \| 75% \| 0.02 \|  \| \| *EWGSOP1* \| 3 \| 192 \| 0.71 (0.34; 0.89) \| 92% \| <0.01 \|  \| \| *EWGSOP2* \| 5 \| 1336 \| 0.31 (0.07; 0.52) \| 96% \| <0.01 \|  \| \| Population \|  \|  \|  \|  \|  \| 0.19 \| \| *Total population* \| 4 \| 1311 \| 0.27 (0.00, 0.50) \| 97% \| <0.01 \|  \| \| *Sarcopenic population* \| 7 \| 376 \| 0.51 (0.21; 0.72) \| 88% \| <0.01 \|  \| \| Age category \|  \|  \|  \|  \|  \| 0.48 \| \| *< 75 years* \| 4 \| 1258 \| 0.54 (0.03; 0.83) \| 96% \| <0.01 \|  \| \| *≥75 years* \| 7 \| 429 \| 0.37 (0.18; 0.53) \| 71% \| <0.01 \|  \| \| Continent \|  \|  \|  \|  \|  \| 0.04 \| \| *Asia* \| 4 \| 609 \| 0.16 (-0.17; 0.46) \| 90% \| <0.01 \|  \| \| *Europe* \| 7 \| 1078 \| 0.55 (0.33; 0.72) \| 98% \| <0.01 \|  \| \| *Correlation type ^1^* \|  \|  \|  \|  \|  \| 0.05 \| \| *Spearman* \| 5 \| 710 \| 0.33 (0.09; 0.52) \| 91% \| <0.01 \|  \| \| *Pearson* \| 3 \| 188 \| 0.72 (0.38; 0.88) \| 91% \| <0.01 \|  \| \| **Social functioning** \|  \|  \|  \|  \|  \|  \| \| Sarcopenia Diagnosis \|  \|  \|  \|  \|  \| 0.79 \| \| *AWGS* \| 4 \| 217 \| 0.42 (0.01; 0.71) \| 90% \| <0.01 \|  \| \| *EWGSOP1* \| 4 \| 279 \| 0.44 (0.34; 0.53) \| 15% \| 0.32 \|  \| \| *EWGSOP2* \| 2 \| 129 \| 0.30 (-0.14; 0.65) \| 85% \| 0.01 \|  \| \| Population \|  \|  \|  \|  \|  \| 0.53 \| \| *Total population* \| 3 \| 24 \| 0.34 (0.10; 0.54) \| 74% \| 0.02 \|  \| \| *Sarcopenic population* \| 7 \| 376 \| 0.44 (0.22; 0.62) \| 82% \| <0.01 \|  \| \| Age category \|  \|  \|  \|  \|  \| 0.20 \| \| *< 75 years* \| 3 \| 167 \| 0.24 (-0.13; 0.55) \| 83% \| <0.01 \|  \| \| *≥ 75 years* \| 7 \| 458 \| 0.48 (0.31; 0.61) \| 74% \| <0.01 \|  \| \| Continent \|  \|  \|  \|  \|  \| 0.90 \| \| *Asia* \| 4 \| 217 \| 0.42 (0.01: 0.71) \| 90% \| <0.01 \|  \| \| *Europe* \| 6 \| 408 \| 0.40 (0.25; 0.52) \| 61% \| 0.02 \|  \| \| *Correlation type ^1^* \|  \|  \|  \|  \|  \| 0.16 \| \| *Spearman* \| 5 \| 339 \| 0.54 (0.42; 064) \| 47% \| 0.11 \|  \| \| *Pearson* \| 3 \| 196 \| 0.27 (-0.14; 0.60) \| 89% \| <0.01 \|  \| \| **Limitation emotional problem** \|  \|  \|  \|  \|  \|  \| \| Sarcopenia Diagnosis \|  \|  \|  \|  \|  \| 0.25 \| \| *AWGS* \| 3 \| 159 \| 0.28 (0.09; 0.45) \| 25% \| 0.26 \|  \| \| *EWGSOP1* \| 4 \| 279 \| 0.42 (0.23; 0.58) \| 71% \| 0.02 \|  \| \| *EWGSOP2* \| 4 \| 886 \| 0.45 (0.35; 0.53) \| 25% \| 0.26 \|  \| \| Population \|  \|  \|  \|  \|  \| 0.59 \| \| *Total population* \| 4 \| 948 \| 0.42 (0.25; 0.57) \| 70% \| <0.01 \|  \| \| *Sarcopenic population* \| 7 \| 376 \| 0.37 (0.27; 0.46) \| 15% \| 0.32 \|  \| \| Age category \|  \|  \|  \|  \|  \| 0.07 \| \| *< 75 years* \| 3 \| 808 \| 0.48 (0.43; 0.53) \| 0% \| 0.43 \|  \| \| *≥ 75 years* \| 8 \| 516 \| 0.37 (0.24; 0.48) \| 56% \| 0.03 \|  \| \| Continent \|  \|  \|  \|  \|  \| 0.14 \| \| *Asia* \| 3 \| 159 \| 0.28 (0.09; 0.49) \| 25% \| 0.26 \|  \| \| *Europe* \| 8 \| 1165 \| 0.43 (0.33; 0.51) \| 53% \| 0.04 \|  \| \| *Correlation type ^2^* \|  \|  \|  \|  \|  \| 0.07 \| \| *Spearman* \| 6 \| 397 \| 0.44 (0.32; 0.55) \| 51% \| 0.07 \|  \| \| *Pearson* \| 2 \| 138 \| 0.26 (0.09; 0.41) \| 0% \| 0.51 \|  \| \| **EQ-5D** \|  \|  \|  \|  \|  \|  \| \| **Self-care** \|  \|  \|  \|  \|  \|  \| \| Sarcopenia Diagnosis \|  \|  \|  \|  \|  \| 0.34 \| \| *AWGS* \| 4 \| 353 \| -0.63 (-0.78; -0.42) \| 84% \| <0.01 \|  \| \| *EWGSOP1* \| 3 \| 192 \| -0.23 (-0.73; 0.42) \| 95% \| <0.01 \|  \| \| *EWGSOP2* \| 6 \| 1471 \| -0.36 (-0.52; -0.18) \| 89% \| <0.01 \|  \| \| Population \|  \|  \|  \|  \|  \| 0.72 \| \| *Total population* \| 6 \| 1525 \| -0.39 (-0.52; -0.25) \| 87% \| <.0.01 \|  \| \| *Sarcopenic population* \| 7 \| 491 \| -0.46 (-0.71; -0.10) \| 93% \| <0.01 \|  \| \| Age category ^3^ \|  \|  \|  \|  \|  \| 0.08 \| \| *< 75 years* \| 4 \| 1313 \| -0.17 (-0.56; 0.27) \| 95% \| <0.01 \|  \| \| *≥75 years* \| 8 \| 603 \| -0.55 (-0.67; -0.39) \| 81% \| <0.01 \|  \| \| Continent \|  \|  \|  \|  \|  \| 0.25 \| \| *Asia* \| 5 \| 803 \| -0.55 (-0.74; -0.27) \| 95% \| <0.01 \|  \| \| *Europe* \| 8 \| 1213 \| -0.35 (-0.55; -0.11) \| 89% \| <0.01 \|  \| \| *Correlation type ^2^* \|  \|  \|  \|  \|  \| 0.21 \| \| *Spearman* \| 10 \| 1133 \| -0.42 (-0.62; -0.18) \| 93% \| <0.01 \|  \| \| *Pearson* \| 1 \| 114 \| -0.58 (-0.69; -0.44) \| - \| - \|  \| \| **Pain/ Discomfort** \|  \|  \|  \|  \|  \|  \| \| Sarcopenia Diagnosis \|  \|  \|  \|  \|  \| **0.81** \| \| *AWGS* \| 5 \| 373 \| -0.23 (-0.37; -0.07) \| 57% \| 0.05 \|  \| \| *EWGSOP1* \| 5 \| 575 \| -0.20 (-0.51; 0.15) \| 90% \| <0.01 \|  \| \| *EWGSOP2* \| 5 \| 696 \| -0.50 (-0.74; -0.14) \| 94% \| <0.01 \|  \| \| Population \|  \|  \|  \|  \|  \| 0.14 \| \| *Total population* \| 7 \| 1209 \| -0.43 (-0.64; -0.17) \| 93% \| <0.01 \|  \| \| *Sarcopenic population* \| 8 \| 435 \| -0.17 (-0.41; 0.09) \| 84% \| <0.01 \|  \| \| Age category ^3^ \|  \|  \|  \|  \|  \| 0.34 \| \| *< 75 years* \| 5 \| 969 \| -0.17 (-0.47; 0.17) \| 90% \| <0.01 \|  \| \| *≥75 years* \| 9 \| 575 \| -0.37 (-0.58; -0.10) \| 89% \| <0.01 \|  \| \| Continent \|  \|  \|  \|  \|  \| 0.53 \| \| *Asia* \| 6 \| 823 \| -0.27 (-0.34; -0.21) \| 48% \| 0.09 \|  \| \| *Europe* \| 9 \| 821 \| -0.37 (-0.60; -0.15) \| 93% \| <0.01 \|  \| \| *Correlation type ^4^* \|  \|  \|  \|  \|  \| 0.87 \| \| *Spearman* \| 11 \| 1381 \| -0.24 (-0.38; -0.10) \| 78% \| <0.01 \|  \| \| *Pearson* \| 1 \| 114 \| -0.26 (-0.42; -0.08) \| - \| - \|  \| \| **Anxiety/Depression** \|  \|  \|  \|  \|  \|  \| \| Sarcopenia Diagnosis \|  \|  \|  \|  \|  \| 0.81 \| \| *AWGS* \| 5 \| 373 \| -0.24 (-0.33; -0.14) \| 7% \| 0.36 \|  \| \| *EWGSOP1* \| 4 \| 279 \| -0.10 (-0.52; 0.35) \| 92% \| <0.01 \|  \| \| *EWGSOP2* \| 6 \| 1394 \| -0.26 (-0.36; -0.14) \| 77% \| <0.01 \|  \| \| Population \|  \|  \|  \|  \|  \| 0.86 \| \| *Total population* \| 7 \| 1612 \| -0.22 (-0.31; -0.13) \| 72% \| <0.01 \|  \| \| *Sarcopenic population* \| 8 \| 434 \| -0.20 (-0.43; 0.05) \| 84% \| <0.01 \|  \| \| Age category ^3^ \|  \|  \|  \|  \|  \| 0.15 \| \| *< 75 years* \| 4 \| 1313 \| -0.01 (-0,39; 0.37) \| 94% \| <0.01 \|  \| \| *≥75 years* \| 10 \| 633 \| -0.30 (-0.37; -0.23) \| 0% \| 0.75 \|  \| \| Continent \|  \|  \|  \|  \|  \| 0.76 \| \| *Asia* \| 6 \| 823 \| -0.19 (-0.30; -0.08) \| 51% \| 0.07 \|  \| \| *Europe* \| 9 \| 1223 \| -0.22 (-0.40; -0.03) \| 82% \| <0.01 \|  \| \| *Correlation type ^2^* \|  \|  \|  \|  \|  \| 0.74 \| \| *Spearman* \| 11 \| 1143 \| -0.22 (-0.37; -0.06) \| 80% \| <0.01 \|  \| \| *Pearson* \| 1 \| 114 \| -0.18 (-0.35; 0.00) \| - \| - \|  \| \| ^1^ 1 study removed (Geerinck,2022) because they did not report the type of correlation used.  ^2^ 2 studies removed (Geerinck, *Matijevic)* because they did not report the type of correlation used. ^3^ 1 study removed (Lee) because they did not report the age of the total population ^4^ 2 studies removed (Geerinck, *Erdogan)* because they did not report the type of correlation used. \| \| \| \| \| \| \| | | | | | | | | | | | | |  |
